# The Efficacy of a Human-Ready mini*MECP2* Gene Therapy in a Pre-Clinical Model of Rett Syndrome

**DOI:** 10.3390/genes15010031

**Published:** 2023-12-24

**Authors:** Chanchal Sadhu, Christopher Lyons, Jiyoung Oh, Indumathy Jagadeeswaran, Steven J. Gray, Sarah E. Sinnett

**Affiliations:** 1 Formerly of Taysha Gene Therapies, Dallas, TX 75247, USA; 2 Formerly of the Department of Pediatrics, University of Texas Southwestern Medical Center (UTSWMC), Dallas, TX 75390, USA; 3 Department of Pediatrics, University of Texas Southwestern Medical Center (UTSWMC), Dallas, TX 75390, USA; 4 Eugene McDermott Center for Human Growth and Development, University of Texas Southwestern Medical Center (UTSWMC), Dallas, TX 75390, USA; 5 Peter O’Donnell Jr. Brain Institute, University of Texas Southwestern Medical Center (UTSWMC), Dallas, TX 75390, USA

**Keywords:** adeno-associated virus (AAV), methyl-CpG binding protein 2 (MeCP2), Rett syndrome (RTT)

## Abstract

Inactivating mutations and the duplication of methyl-CpG binding protein 2 (MeCP2), respectively, mediate Rett syndrome (RTT) and *MECP2* duplication syndrome. These disorders underscore the conceptual dose-dependent risk posed by *MECP2* gene therapy for mosaic RTT patients. Recently, a miRNA-Responsive Autoregulatory Element (miRARE) mitigated the dose-dependent toxicity posed by self-complementary adeno-associated viral vector serotype 9 (AAV9) mini*MECP2* gene therapy (scAAV9/mini*MECP2-myc*) in mice. Here, we report an efficacy assessment for the human-ready version of this regulated gene therapy (TSHA-102) in male *Mecp2*^−/*y*^ knockout (KO) mice after intracerebroventricular (ICV) administration at postnatal day 2 (P2) and after intrathecal (IT) administration at P7, P14 (±immunosuppression), and P28 (±immunosuppression). We also report qPCR studies on KO mice treated at P7-P35; protein analyses in KO mice treated at P38; and a survival safety study in female adult *Mecp2*^−/+^ mice. In KO mice, TSHA-102 improved respiration, weight, and survival across multiple doses and treatment ages. TSHA-102 significantly improved the front average stance and swing times relative to the front average stride time after P14 administration of the highest dose for that treatment age. Viral genomic DNA and mini*MECP2* mRNA were present in the CNS. MiniMeCP2 protein expression was higher in the KO spinal cord compared to the brain. In female mice, TSHA-102 permitted survivals that were similar to those of vehicle-treated controls. In all, these pivotal data helped to support the regulatory approval to initiate a clinical trial for TSHA-102 in RTT patients (clinical trial identifier number NCT05606614).

## 1. Introduction

Rett syndrome (RTT) is an X-linked neurodevelopmental disorder that affects 1 in 10,000 females and is diagnosed less frequently in males [1,2]. RTT is caused by mutations in methyl-CpG binding protein 2 (MeCP2), a transcription regulator whose expression levels must be tightly controlled [3,4]. Female patients with a heterozygous mutation in the *MECP2* gene often survive till the fifth decade of life [5]. Survival in male patients depends on their mutation and karyotype [2].

Key messages can be distilled from pre-clinical *MECP2* gene therapy publications spanning a decade (see the literature summary in Figure S1): first, most publications have shown that *MECP2* gene therapies extend the survival of male *Mecp2*^−/*y*^ (KO) mice [6,7,8,9,10,11,12,13,14,15,16]. Most studies have used adeno-associated virus serotype 9 (AAV9) to deliver a full-length *MECP2* or therapeutic truncated mini*MECP2* gene. Second, unregulated *MECP2* gene therapies have dose-dependent side effects foreshadowed by the literature describing *MECP2* duplication syndrome and animal models of MeCP2 overexpression [7,8,9,11,17]. Depending on the treatment paradigm, side effects in mice can include early death, hindlimb abnormalities, weight loss, or abnormal gait [7,8,9,11]. In humans, *MECP2* duplication syndrome and RTT have many symptoms in common (e.g., intellectual disability and seizures) [4,8]. Third, the greatest survival extensions in KO mice have been observed after neonatal administration, a favorable proof-of-concept age for evaluating pre-clinical gene therapies [6,7,12,17]. In contrast, some researchers have evaluated *MECP2* gene therapies in older, symptomatic KO mice to generate more translationally relevant—albeit more modest—efficacy data from the outset [8,9,17]. Last, certain pre-clinical treatment paradigms, such as those using PHP.B capsids or over-sized viral genomes, are unsuitable for human translation [8,13,14]. 

To address these considerations (e.g., capsid and viral genome size, etc.), researchers designed a regulated AAV9/mini*MECP2-myc* therapy that has been shown to extend KO mouse survival after intrathecal (IT) administration during adolescence [8]. Mini*MECP2* is human-derived, retains conserved regions of *MECP2*, encodes the methyl-binding domain (MBD) and the NCoR/SMRT Interaction Domain (NID) of MeCP2, and covers portions of *MECP2* where many pathogenic mutations occur in male RTT patients [12]. Notably, this viral genome features a novel regulatory element known as miRARE (miRNA-Responsive Autoregulatory Element) [8]. This non-coding element is a panel of miRNA binding sites designed to prevent MeCP2 over-expression-related toxicity in virus-treated mice [8]. Although the mechanistic underpinnings of miRARE have yet to be fully elucidated, in vivo mouse studies have shown that the primary goals of miRARE were achieved. Specifically, miRARE improved the safety of myc-tagged mini*MECP2* gene therapy without compromising its efficacy [8]. 

The next logical step in the consideration of this design as a human treatment is to assess the therapeutic profile of an untagged AAV9/mini*MECP2-*miRARE gene therapy (hereafter named TSHA-102). The neonatal intracerebroventricular (ICV) route was selected to compare outcomes from this current study to previously published studies describing the neonatal administration of *MECP2* gene therapies in mice [6,7,12,15,16]. Although not directly comparable, the IT route was selected for older mice because it bridges published studies with the route selected for an ongoing clinical trial (clinical trial identifier number NCT05606614) [8,18]. 

Frequently published assessments of pre-clinical *MECP2* gene therapies in KO mice are survival extension, weight gain, and aggregate severity score (also known as Bird score) [9,15,16,17]. Weight loss is used as a humane endpoint to mark the date of “death” for KO mouse survival studies [7,8,9,10]. One may hypothesize that weight gain after treatment indirectly reflects improved physiological functions or behaviors (e.g., in RTT patients, breathing, feeding, and gastrointestinal function can impact weight) [19,20,21]. The aggregate severity scoring method visually assesses six phenotypes in RTT mice (see Materials and Methods) [22]. 

In addition to aggregate severity phenotypes, we assessed breathing and gait/limb phenotypes using unrestrained whole-body plethysmography (WBP) and TreadScan, respectively, over a longer examination time. In addition to complementing the Bird score, these orthogonal tests were motivated by the previously published genotype-dependent expressions of miniMeCP2-myc in the midbrain and pons, which help to mediate motor control and breathing, among other functions [8,23,24]. The additional tests also address gaps in the literature. Few pre-clinical RTT gene therapy publications have generated breathing data directly from hosts (see Matagne et al. for conclusive plethysmography results after a single treatment age in KO mice and adult heterozygous female mice [10,11]). Moreover, to our knowledge, no published RTT gene therapy study has extracted breathing sub-scores from aggregate severity scores [6,7,8,9,12,13,14,15,16,25]. In other words, the subjective assessment of breathing phenotypes in KO mice has been assumed but not demonstrated in most RTT gene therapy publications in the past decade. 

An additional cohort of immunosuppressed KO mice were evaluated to further strengthen the study design within the KO mouse model. Daily intraperitoneal (IP) injections of cyclosporine (CsA) have been shown to attenuate lesions and extend survival in older KO mice that would otherwise be at risk of experiencing an immune response to exogenous MeCP2 [13]. The primary motivation for the immunosuppressed arm (+CsA) was to determine if TSHA-102-mediated survival efficacy after administration in older mice (not neonates) was reproducible [13,26]. The immunosuppression regimen described herein is the only one previously published for KO mice receiving *MECP2* gene therapy [13]. 

In KO mice, TSHA-102 improved respiration, weight, and survival across multiple doses and treatment ages. TSHA-102 improved the stance and swing times relative to the total stride time after P14 administration of the highest dose for that treatment age. Viral vector genomic DNA and mini*MECP2* mRNA were present in the CNS. Protein expression was higher in the KO spinal cord compared to the brain. In female mice, TSHA-102 permitted survivals that were similar to those of vehicle-treated controls. 

## 2. Materials and Methods

### 2.1. Animals

Except where otherwise noted, *Mecp2*^−/*y*^ KO mice (B6.129P2(C)-*Mecp2^tm1.1Bird^*/J, stock #3890) were bred at the Jackson Laboratory (JAX) testing site in Maine. To decrease the risk of cannibalism, ICV treatments were administered to pups delivered by experienced dams. The first litters from these dams were used to demonstrate injection proficiency. Only one dam was used per breeder cage to decrease the risk of lateral vector (TSHA-102) transmission. Dams were euthanized after nursing and weaning the treated pups. All mice tested at JAX were male.

Supporting experiments conducted at UTSWMC evaluated: (1) the biodistribution and gene expression in WT and KO mice treated at P30-35; (2) the protein expression in male KO mice treated IT at P38; and (3) safety in female *Mecp2*^−/+^ mice treated IT at six months of age. These mice were from the same strain (JAX stock #3890). 

### 2.2. Blinding and Randomization

Injections, veterinary assessments, and phenotypic assessments at JAX were conducted blind to treatment and genotype, with one exception: researchers were not blind to genotype for the protein expression study (only one genotype was used to quantify protein expression). The genotype of older symptomatic KO mice can sometimes be inferred through visual inspection. Neonates at JAX were shuffled and reassigned to dams so that each dam raised pups that may or may not have been their direct offspring. Neonates were reassigned to dams so that each dam raised the same number of pups (±one mouse). The large-scale breeding program (six breeding rounds with 40 female breeders per round) for the IT study at JAX allowed mice from multiple litters to be assigned to each group. 

### 2.3. Vector

A large-scale lot was produced at Catalent and used to conduct studies at JAX (Figure S2). Self-complementary AAV9/MeP426-mini*MECP2*-miRARE-RDH1pA (referred to as TSHA-102) was prepared in dPBS (137 mM sodium chloride, 8.1 mM sodium phosphate dibasic, 1.47 mM potassium dihydrogen phosphate, and 2.7 mM potassium chloride) containing 5% D-sorbitol and 0.001% Poloxamer 188, pH 7.4 ± 0.1. The manufacturing process involved a triple plasmid transfection of suspension-adapted human embryonic kidney epithelial cells (HEK293F cells) to generate recombinant AAV9 particles [27]. The transgene is an untagged analog of the regulated myc-tagged mini*MECP2* construct [8]. WT AAV serotype 2 inverted terminal repeat (ITR) sequences were modified so that they no longer had Xma1 sites (in contrast to those previously published) [6,8,9]. The dose levels for the JAX study were based on digital droplet PCR (ddPCR) titer results. Quality assessments were conducted before experimental injections at JAX: (1) JAX independently confirmed the sterility of test articles (vehicle and vector); and (2) researchers at UTSW independently re-titered the virus from Catalent via qPCR. 

The same vector lot from Catalent was used for supporting the safety, biodistribution, and gene expression studies conducted at UTSWMC. A research-grade lot produced at the UTSWMC Translational Gene Therapy Core and titered using qPCR was used for the protein expression study (Figure S2). 

### 2.4. Injections

Researchers used dye to confirm their injection proficiencies in a separate cohort of age-matched mice before beginning the experimental injections of TSHA-102 or the vehicle. 

With few exceptions, the injections at JAX were batched according to treatment age. For example, all P2 injections were completed in one week, followed by P28 injections, etc. The ICV injection volume administered at P2 was 1 µL/ventricle (2 µL total). Lumbar IT injection volumes were as follows for each treatment age: P7, 5 µL; P14, 5 µL; and P28, 10 µL. An injection of undiluted virus yielded a maximum dose of 4.4 × 10^11^ vg/mouse in P7 and P14 mice and 8.8 × 10^11^ vg/mouse in P28 mice. The blinded vehicle treatment was dPBS containing 5% D-sorbitol and 0.001% Poloxamer 188, pH 7.4 ± 0.1. CsA was injected IP daily at 5 mL/kg, 10 mg/kg. The injectable CsA was prepared as follows: first, CsA powder (Sigma, PHR1092, St. Louis, MO, USA) was used to prepare a 10 mg/mL stock solution in 650 mg/mL of Cremophor EL and 33% ethanol in sterile saline and stored at 4 °C. Before the IP injections, the stock solution was diluted 1:5 in sterile saline. A subset of conditions was evaluated in the presence of daily CsA among the KO mice: vehicle, 4.4 × 10^11^ vg/mouse, and 8.8 × 10^11^ vg/mouse after P28 administration; and vehicle and 4.4 × 10^11^ vg/mouse after P14 administration. Throughout this manuscript, vehicle administration is implied in all graphs and tables where a vector dose is not specified.

For the supporting biodistribution and mRNA expression study, IT administration was used to deliver 8.8 × 10^11^ vg/mouse to female WT mice, male WT mice, and male KO mice (10 µL); and vehicle to female WT and male KO mice at P30–P35 (10 µL). 

For the supporting protein study, 8.8 × 10^11^ or 2.2 × 10^11^ vg/mouse (10 µL) were administered via IT to KO mice at P38. 

For the supporting female safety study, 8.8 × 10^11^ or 2.9 × 10^11^ vg/mouse (10 µL) or the vehicle were administered via IT to *Mecp2*^−/+^ mice at 6 months of age. 

### 2.5. Weighing

**Figure 1:**
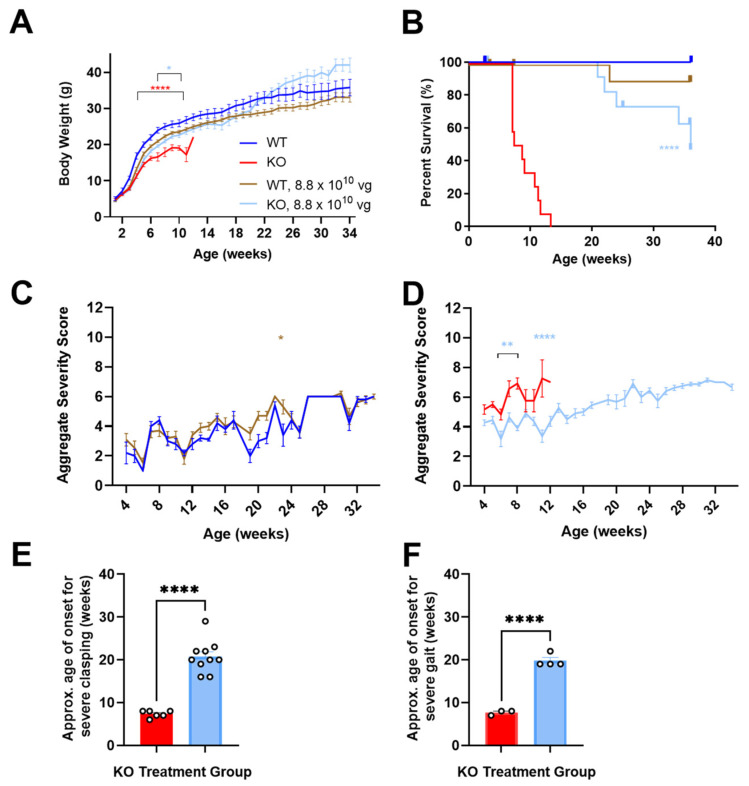
After P2 ICV administration, TSHA-102 is effective in KO mice. (**A**) TSHA-102 improves KO body weight. Statistical analyses were conducted on KO groups through 11 weeks of age and on WT groups up through 34 weeks of age. (**B**) TSHA-102 significantly extends KO survival. TSHA-102-treated KO survival is not significantly different from vehicle-treated WT survival. (**C**) TSHA-102 is well-tolerated in WT mice, with a significant difference in behavior noted at only one time point. (**D**) TSHA-102 improves post-hoc aggregate severity scores in KO mice at the indicated time points. TSHA-102 delays the onset age of (**E**) severe hindlimb clasping and (**F**) severely abnormal gait among KO mice achieving these phenotypes. (**A**–**F**) Color-coding in the figure legend for **A** applies to (**A**–**F**). For example, all red lines and bars describe vehicle-treated KO mice. Levels of statistical significance for this and other figures are defined in the Materials and Methods. Throughout this manuscript, vehicle administration is implied in legends where a vector dose is not specified. (**A**,**C**–**F**) Data are mean ± SEM. *n* per group: (**A**) 6–14; (**B**) 13–14; (**C**) 5–12; (**D**) 11–12; and (**E**,**F**) 10–12. * *p* ≤ 0.05; ** *p* ≤ 0.01; and **** *p* ≤ 0.0001.

Mice were weighed at least once a week except for weeks 35–36 in Figure 1A,B. Mice were provided with DietGel 76A and monitored more frequently once they lost 10% of their peak weight. Female *Mecp2*^−/+^ mice weighing > 36.2 g and <18.1 g pre-injection were excluded from enrollment. 

### 2.6. Aggregate Severity Phenotyping

Methods for aggregate severity score phenotyping have been published [22]. The aggregate severity score combines sub-scores for mobility, gait, hindlimb clasping, tremors, breathing, and general condition. The mobility sub-score describes spontaneity of movement, and the gait sub-score describes stance, waddling, and bunny hops during walking [22]. Each phenotype is scored on a scale from 0 (like WT) to 2 (severe phenotype). The maximum possible aggregate score is 12. Improvement in phenotypes described herein refers to a decreased aggregate score. This scoring system is conducted through visual inspection and requires no equipment. 

### 2.7. Unrestrained Whole-Body Plethysmography (WBP)

Respiratory patterns were recorded in FinePointe version 2.9.212849 software at 7 weeks of age (Buxco FinePointe Whole Body Plethysmography 4-Site System; Buxco Research Products of Data Sciences International, St. Paul, MN, USA). This testing occurred 3–6 weeks after IT injection, depending on injection age. Each test comprised 20 min of acclimation followed by 30 min of testing. Apneas were defined as breathing cycles more than twice the average breathing cycle duration in a sliding 60 s scoring interval, and greater than 0.5 s.

### 2.8. Treadmill

A TreadScan System (CleverSys, Inc., Reston, VA, USA) was used to analyze gait. Individual mice were placed onto a motorized treadmill belt. The motor was turned on and slowly increased to 16.7 cm/s. A camera positioned below the clear belt recorded the gait of the mouse for 20 s. Paw prints were recorded and analyzed. Data for left and right forepaws were averaged; similarly, data for left and right hind paws were averaged. 

### 2.9. Survival

Across published studies, death has been defined as humane euthanasia after mice lose 20% of their peak weight; veterinarian-requested euthanasia; and/or natural death [6,8,9,13]. At JAX, veterinarians requested euthanasia for the following conditions: severe tail lesions, paraphimosis, head tilt, toe injury, fight wounds, and hydrocephaly [28]. This level of detail for veterinarian-requested euthanasia is typically not available in publications, but we are disclosing this for transparency. At JAX, Kaplan–Meier curves and median survival values were obtained after censoring (not excluding) mice that were euthanized for the above conditions. Mice that failed to thrive (possibly due to poor maternal care) up to age P27 were also censored in Kaplan–Meier analyses. 

### 2.10. End-of-life Biodistribution and Gene Expression

Tissue generated at JAX was collected from vehicle- and TSHA-102-treated mice at the time of euthanasia, stored at <−60 °C, and then shipped on dry ice to Northern Biomolecular Services (NBS). Cerebella were dissected separately from the rest of the brain tissue originating from JAX. Mice found dead were excluded from analyses. Samples were stored at <−60 °C until nucleic acid extraction. NBS used a QuantStudio 7 Flex Real Time PCR system (Applied Biosystems, Waltham, MA, USA). Primers for mini*MECP2* were 5′- CCC AAG AAA AAG CGG AAG GT-3′ (forward) and 5′-TCT TGA TGG GGA GTA CGG TC-3′ (reverse). The probe was 5′-6FAM-CCG GGG AGT GTG GTG-MGBNFQ-3′. For biodistribution, results of <5 copies of double-stranded TSHA-102 vector DNA per qPCR reaction were reported as being below the limit of detection (BLOD). Results of ≥5 and <25 copies were reported as being below the limit of quantification (BLOQ). For gene expression, NBS used an iTaq Universal Probe One-Step Master Mix and iScript Reverse Transcriptase (Bio-Rad, Hercules, CA, USA). For gene expression, results of <10 copies of single-stranded vector-derived mRNA per 100 ng of total RNA were reported as BLOD; ≥10 and <100 copies were reported as BLOQ. Vehicle controls revealed no biodistribution or expression of TSHA-102. Hypoxanthine phosphoriboxyltransferase 1 (*Hprt1*) mRNA Ct values confirmed the integrity of the extracted RNA. Ct values from no reverse transcriptase treatment controls were used to monitor DNA contamination.

Studies using tissues from UTSWMC were conducted similarly with the following exceptions: (1) brain samples included cerebella; and (2) NBS calculated expression means by assigning BLOQ data a value of 100 ss copies/µg RNA (in contrast, NBS assigned BLOQ data a value of 5 ss copies/µg RNA in the final report for JAX tissue). Thus, the two original qPCR reports are not directly comparable. 

### 2.11. Immunofluorescence

Tissue sectioning and immunofluorescence analyses for the treated KO mice were conducted as previously described [8]. Mice were perfused 3 weeks after injection with 4% paraformaldehyde (PFA; Thermo catalog # J19943.K2, Waltham, MA, USA). Dissected tissue was then drop-fixed for 2–5 days (brain) or 2–3 weeks (spinal cord) at 4 °C before sectioning. An antigen retrieval step at 90 °C with 10 mM sodium citrate (pH 6) was included before blocking [8]. The immunofluorescence study used an anti-MeCP2 primary antibody (Sigma catalog # WH0004204M1, St. Louis, MS, USA) and anti-mouse secondary antibody (Invitrogen catalog # A32723, Waltham, MA, USA). The primary antibody recognizes an epitope encoded by exons 3–4 of full-length endogenous mouse and human *MECP2* and human-derived mini*MECP2*. The B6.129P2(C)-*Mecp2^tm1.1Bird^*/J KO strain is missing exons 3–4 of endogenous *MECP2* [29]. Age-matched controls were examined prior to quantitative analyses: (1) a KO negative control (no anti-MeCP2 primary antibody) qualitatively confirmed low background; and (2) a WT positive control for endogenous MeCP2 expression in a *Mecp2*^+/*y*^ mouse (treated with AAV9/*FOXG1-myc* for an unrelated study) confirmed the successful application of the primary antibody. 

### 2.12. Confocal Microscopy

Microscopy was conducted as previously described [8], with the following exceptions: anti-miniMeCP2 signal was examined in 5 images per tissue region per KO mouse, with 3–5 KO mice per group. For brain regions in which no miniMeCP2 signal was observed, all of the sections on the microscope slides were examined to ensure a correct conclusion. Tile scans were recorded with a 10× objective and 2× digital zoom. Close-up images were recorded with a 20× objective with 2× digital zoom.

### 2.13. Statistics

Software: GraphPad Prism 8 and 9 were used to calculate significance. The threshold for significance was defined as *p* ≤ 0.05. Throughout, the following convention is used to convey significance: * *p* ≤ 0.05; ** *p* ≤ 0.01; *** *p* ≤ 0.001; and **** *p* ≤ 0.0001. Post-hoc adjusted *p* values are reported when ANOVAs are used. Throughout, weight and aggregate severity figures list the *n* per group at the start of data collection. Data, excluding tables, show means ± SEM. 

Weight and aggregate severity scores: Two-way ANOVAs followed by Sidak’s multiple comparison test were conducted at time points for which 2 or more mice were alive in each group when comparing vehicle- versus virus-treated KO mice; when comparing vehicle- or virus-treated WT mice; and when comparing vehicle-treated WT and KO groups. 

Survival: The Gehan–Breslow–Wilcoxon test assessed the significance between vehicle- and virus-treated mice [6,8,9]. 

WBP and TreadScan: Except where otherwise noted, a one-way ANOVA followed by Dunnett’s multiple comparisons test was conducted to evaluate statistical differences between the vehicle- and virus-treated KO control group. A *t*-test was conducted to evaluate statistical differences between the vehicle-treated controls. 

Phenotype age of onset: For the ICV study, a *t*-test was used to evaluate the onset age for severe sub-scores from the aggregate severity scoring system. For the IT study, a one-way ANOVA followed by Dunnett’s multiple comparisons test was used to compare the compatibility of conclusions across plethysmography and severity sub-score readouts. Onset analyses excluded mice that were censored in the survival analyses. 

Biodistribution, gene expression, and protein expression: qualitative descriptions are provided.

## 3. Results

TSHA-102 treatment efficacy results were obtained from male KO mice, as detailed below. 

### 3.1. Neonatally Administered TSHA-102 Improves Body Weight in KO Mice

P2 ICV administration of TSHA-102 increased the KO mice body weight (*p* ≤ 0.05 versus vehicle-treated KO mice; *n* = 13–14 KO mice/group; Figure 1A). For most of the study duration, virus-treated WT mice showed a trend in decreased weight. Their growth followed an otherwise normal trajectory (8.8 × 10^10^ vg/mouse versus vehicle-treated WT mice; *p* ≥ 0.05 when the analysis extends to 34 weeks of age; *p* ≤ 0.05 when the analysis is limited to 11 weeks of age; *n* = 6–12 WT mice/group; Figure 1A) [12]. 

### 3.2. Neonatally Administered TSHA-102 Improves Survival in KO Mice 

P2 ICV administration of TSHA-102 extended median survival by over 300% (*p* ≤ 0.0001 versus vehicle-treated KO mice; *n* = 13–14 KO mice enrolled per group; 2–3 censored mice/group; Figure 1B). Conditions warranting censoring in Kaplan–Meier analyses (e.g., veterinarian-requested euthanasia for lesions) are described in the Materials and Methods. TSHA-102 was well-tolerated in WT mice compared to vehicle-treated WT mice (*p* > 0.05; *n* = 6–12 WT mice enrolled per group; 1–2 censored mice/group).

### 3.3. Neonatally Administered TSHA-102 Improves Aggregate Severity Scores in KO Mice

After P2 ICV administration, no sustained difference in aggregate severity was observed between the vehicle- and virus-treated WT mice (*p* ≤ 0.05 at only one time point; *n* = 5–11 WT mice/group; Figure 1C). In contrast, TSHA-102 improved (decreased) aggregate RTT severity scores in the KO mice at several time points compared to the untreated KO mice (*p* ≤ 0.01; *n* = 11–12 KO mice/group; Figure 1D). 

### 3.4. Neonatally Administered TSHA-102 Delays the Onset of Severely Abnormal Clasping and Gait

After P2 ICV administration, 25% and 33% of the vehicle-treated KO mice developed severely abnormal mobility and conditions, respectively. Half (50%) of the vehicle-treated KO mice developed severe clasping, with an approximate onset age of 7 weeks. In addition, 25% of the vehicle-treated KO mice developed severely abnormal gait, with an approximate onset age of 8 weeks.

In contrast, the TSHA-102-treated KO mice showed near WT-like mobility and condition scores (no severe level 2 scores) and a delayed onset of severely abnormal hindlimb clasping and gait. More specifically, all the TSHA-102-treated KO mice (excluding mice censored in the survival analyses) developed severe clasping later at approximately 21 weeks (*p* ≤ 0.0001, approximately 200% delayed onset for TSHA-102 versus vehicle; *n* = 6–10 KO mice/group developed severe clasping; Figure 1E). In total, 40% of the TSHA-102-treated KO mice developed severe gait later at approximately 20 weeks (*p* ≤ 0.001, approximately 150% delayed onset for virus versus vehicle; *n* = 3–4 KO mice/group developed severely abnormal gait; Figure 1F). The lower percentage (25%) of vehicle-treated KO controls developing severely abnormal clasping and gait may be an artifact of their early deaths. 

Only 1–2 KO mice/group developed level 2 scores for severely abnormal breathing. No significant treatment effect was observed for tremors (*n* = 3 KO mice/group achieving severe tremors, onset age of 7 ± 4 weeks of age for vehicle-treated mice; and 16 ± 8 weeks of age for TSHA-102-treated mice). 

### 3.5. Intrathecal TSHA-102 Improves Weight in Older KO Mice

**Figure 2:**
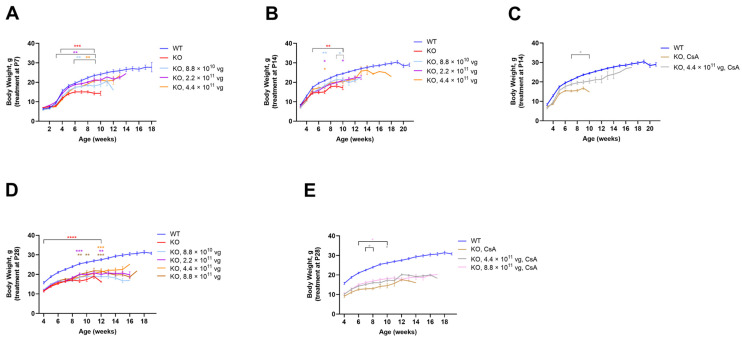
TSHA-102 improves weight in older KO mice. (**A**) After P7 and (**B**) P14 administration, all doses increase KO weight during the indicated timespans. (**C**) After P14 and in the presence of immunosuppression, 4.4 × 10^11^ vg/mouse improves KO weight during the indicated timespan. (**D**) After P28 administration, each dose increases KO weight during the indicated time points. (**E**) After P28 administration and in the presence of immunosuppression, each dose increases KO weight during the indicated time points. (**A**–**E**) Asterisks for virus-treated KO mice are color-matched to their respective group and indicate statistical comparisons against vehicle-treated KO mice (**A**,**B**,**D**). Red asterisks indicate comparisons between vehicle-treated KO mice and vehicle-treated WT mice. (**C**,**E**) A statistical comparison for WT versus immunosuppressed KO mice is not provided because the groups differ by two variables. (**A**–**E**). The same WT group is shown across graphs. Data are mean ± SEM. *n* per group: (**A**,**B**) 10–12; (**C**) 7–12; and (**D**,**E**) 12. * *p* ≤ 0.05; ** *p* ≤ 0.01; *** *p* ≤ 0.001; and **** *p* ≤ 0.0001.

TSHA-102 was administered IT at P7, P14, and P28, with the oldest age representing adolescence. Enrollment for the +CsA study arm was limited to the following KO groups: vehicle and 4.4 × 10^11^ vg/mouse after P14 administration; and vehicle, 4.4 × 10^11^, and 8.8 × 10^11^ vg/mouse after P28 administration. TSHA-102 consistently improved KO weight with effective doses spanning a 10-fold dose range; the effective doses varied across treatment paradigms (Figure 2). The best results were observed when treatment was administered early or in conjunction with CsA. A detailed presentation of the data shown in Figure 2 is provided below.

The vehicle-treated KO mice (±CsA) weighed less than the vehicle-treated WT mice (*p* ≤ 0.01 after P7-P28 administration; *n* = 12 mice per vehicle group; Figure 2). After P7 administration, each dose increased the KO weight over a relatively long period: beginning at 3 weeks of age for the KO mice treated with 2.2 × 10^11^ vg/mouse (*p* ≤ 0.01; *n* = 12 KO mice/group; Figure 2A); and from 6 weeks of age for the KO mice treated with 8.8 × 10^10^ and 4.4 × 10^11^ vg/mouse (*p* ≤ 0.01; *n* = 10–12 KO mice/group; Figure 2A). Although each dose increased weight after P14 administration, the duration of efficacy was brief. For example, the KO mice treated at P14 with 4.4 × 10^11^ vg/mouse showed greater weight versus vehicle-treated KO mice at seven weeks of age (*p* ≤ 0.05; *n* = 12 KO mice/group; Figure 2B). Immunosuppressed mice treated with the same dose at P14 demonstrated a more sustained weight gain from 7 to 10 weeks of age (*p* ≤ 0.05 versus vehicle-treated KO mice; *n* = 7–12 KO group; Figure 2C). Likewise, after P28 administration, immunosuppression provided a more sustained benefit. For example, the highest dose (8.8 × 10^11^ vg/mouse without CsA) increased weight sporadically (*p* ≤ 0.01 versus vehicle-treated KO mice; *n* = 12 KO mice/group; Figure 2D). In contrast, +CsA dose-matched mice showed significantly increased weight from 6 to 10 weeks of age (*p* ≤ 0.05 versus +CsA, vehicle-treated KO mice; *n* = 12 KO mice/group; Figure 2E). 

### 3.6. Intrathecal TSHA-102 Improves Survival in Older KO Mice

Table 1 summarizes the survival analyses for the KO mice treated IT with vehicle or TSHA-102 at P7, P14, or P28. Some mice were censored due to veterinary events (see Materials and Methods).

In the absence of CsA, TSHA-102 significantly extended median survival over at least a four-fold dose range when administered at P7, P14, or P28 (*p* ≤ 0.05; 11–12 KO mice enrolled per group; 0–4 study censored mice/group; Table 1). The longest extension (57% increase compared to untreated KO mice) was observed after P28 administration of TSHA-102 at 8.8 × 10^11^ vg/mouse (without CsA; *p* ≤ 0.01; *n* = 12 KO mice enrolled per group; 0–4 censored mice/group; Table 1).

In the +CsA study arm, significant extensions were observed after the administration of 4.4 × 10^11^ vg/mouse at P14 and 8.8 × 10^11^ vg/mouse at P28 (*p* ≤ 0.01; *n* = 12–13 and 12 KO mice/group, respectively; 1–7 and 0–1 censored mice/group, respectively; Table 1).

Among the KO mice treated with 8.8 × 10^11^ vg/mouse of TSHA-102 at P28 without CsA, seven mice developed wounds. Four of those seven mice were euthanized and censored; none of the dose- and age-matched +CsA KO mice developed wounds. Due to censoring, similar survival extensions were observed after the P28 administration of 8.8 × 10^11^ vg/mouse with and without CsA (Table 1).

### 3.7. Intrathecal TSHA-102 Improves Respiration in Older KO Mice

Respiration, as measured by WBP, was evaluated at 7 weeks of age, a symptomatic young adult stage approaching the median lifespan of the vehicle-treated KO mice. The time between treatment age and testing age varied across the P7, P14, and P28 treatment age groups, so comparisons of efficacy across treatment ages should be interpreted cautiously.

**Figure 3:**
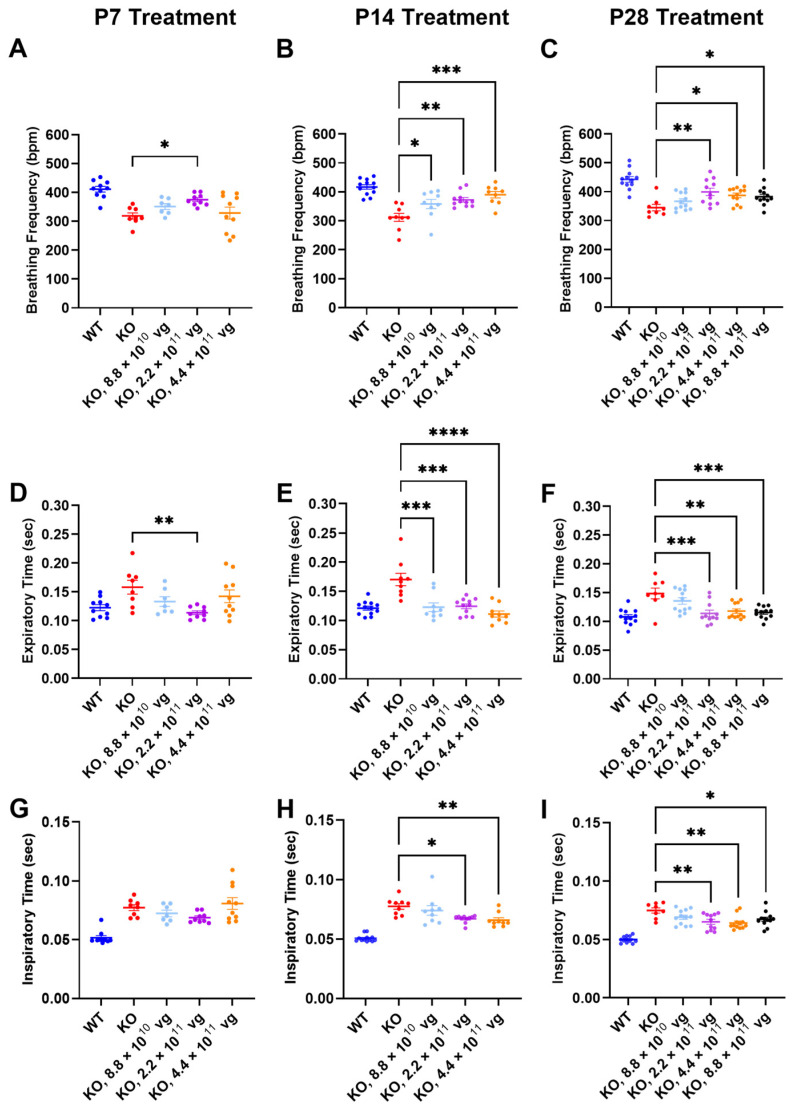
TSHA-102 improves breathing frequency in older KO mice. Although not notated above for simplicity, there was a significant difference between WT and KO vehicle-treated controls for respiratory readouts (*p* ≤ 0.05). After (**A**) P7, (**B**) P14, and (**C**) P28 administration, the indicated doses of TSHA-102 increased breathing frequency. After (**D**) P7, (**E**) P14, and (**F**) P28 administration, the indicated doses of TSHA-102 decreased expiratory time. (**G**) TSHA-102 did not improve inspiratory time after P7 administration. After (**H**) P14 and (**I**) P28 administration, the indicated doses of TSHA-102 decreased inspiratory time. Data are mean ± SEM. *n* per group: (**A**,**D**,**G**) 7–10; (**B**,**E**,**H**) 9–12; and (**C**,**F**,**I**) 8–12. * *p* ≤ 0.05; ** *p* ≤ 0.01; *** *p* ≤ 0.001; and **** *p* ≤ 0.0001.

Compared to vehicle-treated KO mice, TSHA-102 significantly increased the breathing frequency in KO mice treated at P7, P14, and P28. The effective dosing range (two- to five-fold) for treatment at P14 and P28 was greater than that observed for P7. Across treatment ages, the lowest dose was least effective (P7: 2.2 × 10^11^ vg/mouse, *p* ≤ 0.05, *n* = 8–9 KO mice/group; P14: 8.8 × 10^10^–4.4 × 10^11^ vg/mouse, *p* ≤ 0.05, *n* = 9–11 KO mice/group; and P28: 2.2 × 10^11^–8.8 × 10^11^ vg/mouse, *p* ≤ 0.05, *n* = 8–12 KO mice/group) (Figure 3A–C).

The improved breathing frequency in the TSHA-102-treated KO mice was due to decreased expiratory and inspiratory time. Compared to vehicle-treated KO mice, TSHA-102 significantly decreased the expiratory time in the KO mice treated at P7, P14, and P28 (P7: 2.2 × 10^11^ vg/mouse, *p* ≤ 0.01, *n* = 8–9 KO mice/group; P14: 8.8 × 10^10^–4.4 × 10^11^ vg/mouse; *p* ≤ 0.001, *n* = 9–11 KO mice/group; and P28: 2.2 × 10^11^–8.8 × 10^11^ vg/mouse; *p* ≤ 0.01, *n* = 8–12 KO mice/group) (Figure 3D–F). TSHA-102 had no effect on inspiratory time after P7 administration in the KO mice. Compared to the vehicle-treated KO mice, TSHA-102 significantly decreased the inspiratory time in the KO mice treated at P14 and P28 (P14: 2.2 × 10^11^–4.4 × 10^11^ vg/mouse, *p* ≤ 0.05, *n* = 9–11 KO mice/group; P28: 2.2 × 10^11^–4.4 × 10^11^ vg/mouse, *p* ≤ 0.05, *n* = 8–12 KO mice/group) (Figure 3G–I).

**Figure 4:**
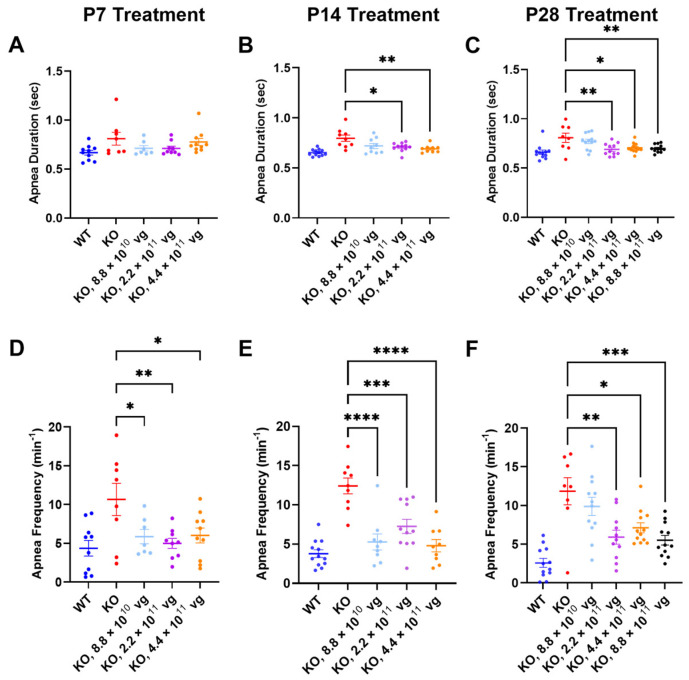
TSHA-102 improves apnea phenotypes in older KO mice. Although not notated above for simplicity, there was a significant difference between WT and KO vehicle-treated controls for respiratory readouts (*p* ≤ 0.05). (**A**) After P7 administration, TSHA-102 did not affect apnea duration. After P14 (**B**) and P28 (**C**) administration, the indicated doses of TSHA-102 decreased apnea duration. After P7 (**D**) and P14 (**E**) administration, each dose of TSHA-102 decreased apnea frequency. (**F**) After P28 administration, the indicated doses of TSHA-102 decreased apnea frequency. The lowest dose was not effective at treating apnea phenotypes after P28 administration. Data are mean ± SEM. *n* per group: (**A**,**D**) 7–10; (**B**,**E**) 9–12; and (**C**,**F**) 8–12. * *p* ≤ 0.05; ** *p* ≤ 0.01; *** *p* ≤ 0.001; and **** *p* ≤ 0.0001.

Compared to the vehicle-treated KO mice, TSHA-102 improved one or more apnea parameters over a two- to five-fold dose range across treatment ages. Specifically, TSHA-102 decreased the apnea duration in the KO mice treated at P14 and P28, but not in the KO mice treated at P7 (P14: 2.2 × 10^11^–4.4 × 10^11^ vg/mouse; *p* ≤ 0.05, *n* = 9–11 KO mice/group; P28: 2.2 × 10^11^–8.8 × 10^11^ vg/mouse; *p* ≤ 0.05, *n* = 8–12 mice/group) (Figure 4A–C). Compared to the vehicle-treated KO mice, all doses of TSHA-102 decreased the apnea frequency in KO mice (P7: 8.8 × 10^10^–4.4 × 10^11^ vg/mouse; *p* ≤ 0.05; *n* = 7–8 KO mice/group; P14: 8.8 × 10^10^–4.4 × 10^11^ vg/mouse; *p* ≤ 0.001; *n* = 9–11 KO mice/group; and P28: 2.2 × 10^11^–8.8 × 10^11^ vg/mouse; *p* ≤ 0.05; *n* = 8–12 KO mice/group) (Figure 4D–F).

### 3.8. Daily Immunosuppression May Improve Respiration in Older Vehicle-Treated KO Mice

Only a sporadic efficacy for 4.4 × 10^11^ vg/mouse (+CsA) after P28 administration was observed, and the significance was consistent with that in the non-immunosuppressed study arm (Table S1). Most of the respiratory readouts in Table S1 showed no efficacy for the three TSHA-102-treated KO groups receiving daily CsA (Table S1). Among the P14 and/or P28 treatment groups, CsA alone appeared to improve apnea duration, apnea frequency, breathing frequency, inspiratory time, and expiratory time (*p* ≤ 0.05 comparing vehicle-treated KO mice with and without CsA; *n* per KO group is 9–11 mice for P14 administration and 8–12 mice for P28 administration; Figure S3). An improved respiratory phenotype for +CsA vehicle-treated KO mice (versus −CsA vehicle-treated KO mice) precluded a more robust demonstration of efficacy for TSHA-102.

Alternative ANOVA structuring aggregating all KO groups within each treatment age (IT vehicle and all doses of IT TSHA-102, ±CsA) likewise indicated that the CsA treatment regimen provided a significant therapeutic effect for expiratory time after P14 administration; and apnea frequency, apnea duration, and expiratory time after P28 administration.

### 3.9. Aggregate Severity Scores Did Not Reveal Compelling Behavioral Efficacy in Older KO Mice

Table 2 summarizes the frequency of significant post-hoc comparisons for the aggregate phenotype severity of the vehicle- versus TSHA-102-treated KO mice. Most scores showed no significant post-hoc difference between vehicle and TSHA-102 treatments. Although sporadic differences were observed at isolated time points, a visual assessment of the data reveals overlapping curves (Table 2, Figure S4). Among the mice treated at P7, for example, post-hoc comparisons showed a significant change (decrease in severity) at 1–2 time points per dose (*p* ≤ 0.05; *n* = 10–12 KO mice/group; immunosuppressed P7 treatment groups were not enrolled; Table 2). Notably, this significance consistently occurred at 6 weeks of age, when there was a downward spike in the aggregate score (Figure S4). This unusual data signature (a spike punctuating a smooth curve) warrants a cautious interpretation. The downward spike is not an artifact of death, as the median survival among groups treated at P7 exceeded 6 weeks of age. CsA did not impact the aggregate scores after TSHA-102 administration at P14 and P28 for the indicated doses (Table 2).

### 3.10. Limited Efficacy in Older KO Mice in Selected Phenotype Severity Sub-Scores

We have previously extracted Bird sub-scores for selected phenotypes (i.e., gait and clasping) to further elucidate efficacy or toxicity [8,9]. Because severe phenotypes can be visually assessed more reliably than mild phenotypes, the approximate average onset age for severe (sub-score = 2) phenotypes was analyzed. Comparisons across treatment ages for P2 versus P7-P28 should be described cautiously, because multiple parameters (e.g., route, dose, and injection volume) vary in these comparisons. Table S2 summarizes statistical comparisons for extracted sub-scores for mobility, gait, clasping, tremors, breathing, and general condition, in the absence of CsA. Key observations are described below.

**Figure 5:**
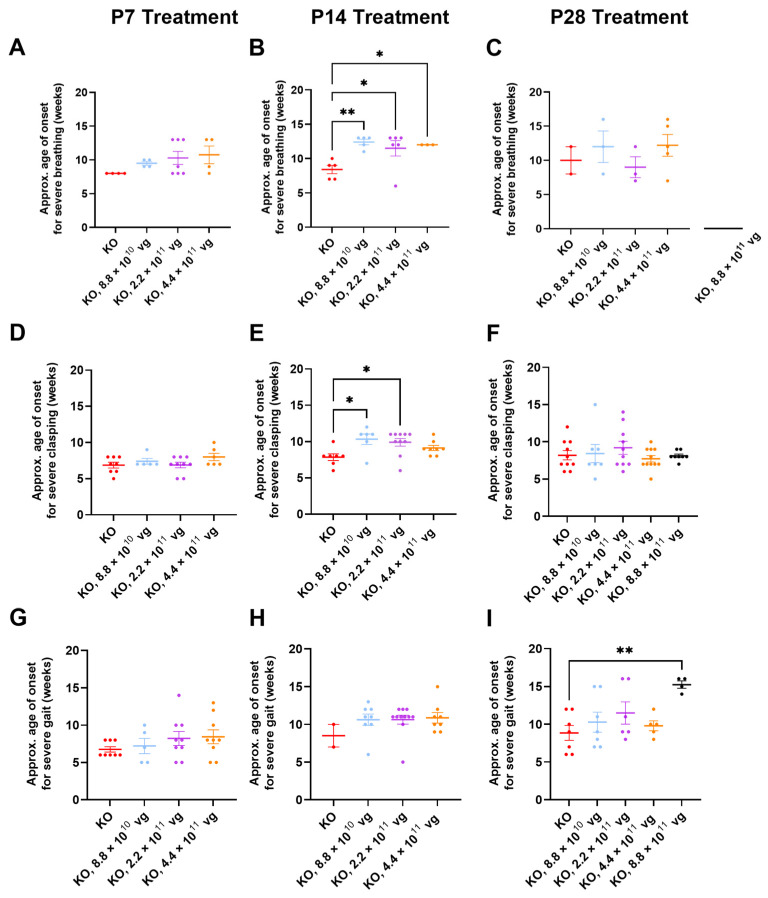
TSHA-102 delays the onset of generalized RTT-like phenotypes in KO mice in a subset of treatment paradigms. The approximate onset ages for severe (**A**–**C**) breathing, (**D**–**F**) clasping, and (**G**–**I**) gait were quantified. (**A**–**C**) Few vehicle-treated KO mice achieved level 2 scores for abnormal breathing. (**B**) Virus-treated mice experienced a 48% delayed onset of severely abnormal breathing after P14 administration (*p* ≤ 0.05). (**C**) No mice treated with the highest dose at P28 developed severe breathing scores (see broken X axis). (**E**) After treatment at P14, virus-treated mice demonstrated an approximately 30% delay in the onset age for severe clasping (8.8 × 10^10^ vg/mouse; compared to the approximately 200% delay after dose-matched neonatal ICV administration). (**I**) After P28 administration, mice treated with the highest dose experienced an approximately 70% delay in developing severely abnormal gait. Data are mean ± SEM. *n* developing severe scores per group: (**A**) 4–7; (**B**) 3–6; (**C**) 2–5; (**D**) 5–9; (**E**) 6–10; (**F**) 7–11; (**G**) 5–9; (**H**) 2–11; and (**I**) 4–7. * *p* ≤ 0.05; ** *p* ≤ 0.01.

Few vehicle-treated KO mice (2–5 mice per treatment age without CsA) achieved level 2 sub-scores for breathing during their lifespan (Figure 5A–C). This result contrasts with WBP data showing abnormal respiration for many saline-treated KO mice (Figure 3 and Figure 4). Thus, the conventional Bird scoring approach may preclude efficacy detection, simply because the control data are not reliably robust.

Subjective generalized scoring detected a delayed onset of severely abnormal breathing in virus-treated mice after P14 administration (*p* ≤ 0.05, vehicle versus each of three doses post-hoc; *n* = 3–6 KO mice/group developed level 2 scores; Figure 5B). A similar percentage of vehicle- and TSHA-102-treated KO mice (8.8 × 10^10^ vg/mouse) developed severe breathing scores after P14 administration (45–50%, *n* = 5 KO mice per each of 2 groups developed level 2 scores). KO mice treated with the lowest dose (8.8 × 10^10^ vg/mouse) had a 48% delayed onset for severely abnormal breathing (versus vehicle-treated KO mice). The longer survival of the virus-treated group lends more confidence to the interpretation, as early deaths would preclude the collection of level 2 data points.

Clasping sub-scores were extracted to compare with the neonatal cohort data. Over half of both vehicle- and virus-treated KO mice achieved severe clasping scores after treatment at P14 (8.8 × 10^10^ and 2.2 × 10^11^ vg/mouse; Figure 5E). Virus-treated KO mice with severe clasping developed their level 2 scores with a 30% delayed onset (8.8 × 10^10^ vg/mouse versus vehicle; *n* = 6–7 KO mice developed level 2 scores, Figure 5E). This efficacy is modest compared to the approximately 200% delay after neonatal ICV administration (Figure 1E and Figure 5E). No efficacy for clasping was observed after treatment at P7 and P28 (Figure 5D,F).

Gait sub-scores were extracted to indirectly compare with the neonatal data. KO mice treated with the highest dose at P28 experienced an approximately 70% delay in developing severely abnormal gait (*p ≤* 0.01, *n* = 4–7 KO mice developed level 2 scores). This efficacy is modest compared to the approximately 150% delay after neonatal ICV administration of a 10-fold lower dose (Figure 1F and Figure 5I). The observed delay in severe gait onset was reproducible in the +CsA study arm (P28 administration, 8.8 × 10^11^ vg/mouse, 52% delay, *p* ≤ 0.05, *n* = 8–11 KO mice/group, Table S3). Moreover, the delay observed in the absence of CsA is consistent with the 50% delay in developing severely abnormal gait for KO mice treated IT with a high dose of a myc-tagged version of TSHA-102 (few virus-treated mice developed abnormal gait in that study) [8]. No efficacy for delaying the onset of abnormal gait was observed after treatment at P7 and P14 in the absence of CsA (Figure 5G,H).

Table S3 summarizes statistical comparisons for the extracted sub-scores for mobility, gait, clasping, tremors, breathing, and general condition in the presence of CsA. Aside from gait analyses after P28 administration, no significant differences were observed for most of the phenotypes and comparisons.

### 3.11. Intrathecal TSHA-102 Significantly Changes Abnormal Gait Phenotypes in KO Mice Treated at P14

**Figure 6:**
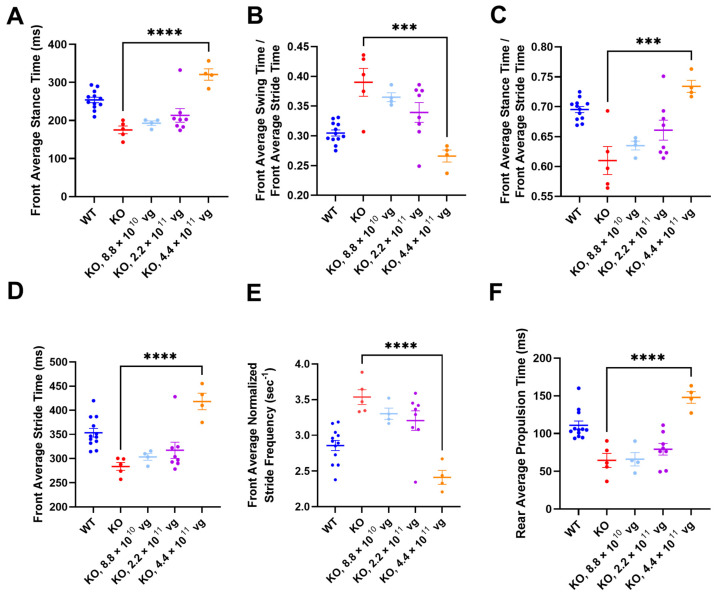
TSHA-102 significantly changes gait parameters in KO mice after P14 administration. For simplicity, significant differences are notated for comparisons between KO groups only. The same dose (4.4 × 10^11^ vg/mouse) significantly changed 6 abnormal gait phenotypes: (**A**) front average stance time, (**B**) front average swing time/front average stride time, (**C**) front average stance time/front average stride time, (**D**) front average stride time, (**E**) front average normalized stride frequency, and (**F**) rear average propulsion time. Data are mean ± SEM. *n* per group is 4–12. *** *p* ≤ 0.001; and **** *p* ≤ 0.0001.

TreadScan was used to evaluate gait parameters for all enrolled IT groups. Among vehicle KO controls treated at P7, only one mouse completed TreadScan testing, thereby precluding efficacy comparison (Figure S5A,E,I,M,Q,U). After P14 administration (without CsA), TSHA-102 at 4.4 × 10^11^ vg/mouse significantly increased front average stance time, front average stride time, rear average propulsion time, and the ratio of front average stance time to front average stride time, and significantly decreased the front average normalized stride frequency and the ratio of the front average swing time to the front average stride time (versus vehicle-treated KO mice; *p* ≤ 0.001; *n* = 4–5 ambulatory mice per KO group; Figure 6).

Alternative ANOVA structuring comparing WT mice versus all other groups showed no significant difference between WT controls and vector-treated KO mice (4.4 × 10^11^ vg/mouse) for the ratios of the front average swing time to the front average stride time, and of the front average stance time to the front average stride time (*n* = 4–12 ambulatory mice per group; Figure 6B,C). The same alternative ANOVA structuring showed a significant difference between WT controls and vector-treated KO mice (4.4 × 10^11^ vg/mouse) for the remaining parameters (*p ≤* 0.05; *n* = 4–12 ambulatory mice per group; Figure 6A,D–F). A conservative interpretation of this data is that TSHA-102 is on the right track for improving gait parameters (i.e., TSHA-102 increases abnormally low measurements and decreases abnormally high measurements) and that further refinement of the treatment may be warranted. WT and KO control groups treated with vehicle at P14 had significantly different gait parameters (*p* ≤ 0.001; *n* = 5–12 ambulatory mice per vehicle-treated group; Figure 6).

The TreadScan readouts did not show a consistently significant difference between vehicle-treated controls after P28 administration (Figure S5C,G,K,O,S,W). The means for the vehicle-treated controls were similar (WT versus KO) for each of the six TreadScan readouts (without CsA; *n* = 4–12 ambulatory mice per vehicle-treated group; *p* > 0.05 rear average propulsion time and the ratios of the front average stance time to the front average stride time, and of the front average swing time to the front average stride time; *p* ≤ 0.05 with a narrow difference between the means for control groups for the front stance time, front stride time, and normalized stride frequency; Figure S5C,G,K,O,S,W). The narrow differences between control groups may have precluded efficacy detection (*p* > 0.05 for vehicle-versus TSHA-102-treated KO mice, for each of six TreadScan readouts, after P28 administration without CsA; *n* = 4–11 ambulatory KO mice/group; Figure S5C,G,K,O,S,W).

The +CsA study arm comprised two vehicle-treated KO groups and three TSHA-102-treated KO groups, spanning the P14 and P28 treatment ages (Figure S5). No statistics were performed on the +CsA mice treated at P14 because only 1–2 ambulatory KO mice/group completed TreadScan testing (Figure S5B,F,J,N,R,V). After P28 administration in the presence of CsA, the baseline phenotype of the vehicle-treated KO mice could not be assessed because the +CsA arm of the study did not include a vehicle-treated WT control group (Figure S5D,H,L,P,T,X). Among the six TreadScan phenotypes examined, a significant difference was observed the between vehicle- and TSHA-102-treated (8.8 × 10^11^ vg/mouse) KO mice for rear average propulsion time only (*p* ≤ 0.001, *n* = 6–11 ambulatory KO mice/group; Figure S5X). No significant differences were observed for the remaining five TreadScan phenotypes examined among the immunosuppressed KO mice.

### 3.12. Biodistribution Analyses Confirmed Delivery of TSHA-102 to the CNS across Treatment Ages (P7-P35), Genders, and Genotypes

Scheduled (4 weeks post-injection) and end-of-life tissue samples were collected at UTSW and JAX for qPCR, respectively (Figures S6 and S7). The results are discussed qualitatively without statistics. Table S4 lists the lifespans of mice selected for end-of-life assessment. Across the study sites, all biodistribution data for the TSHA-102-treated mice were quantifiable. Biodistribution results are not directly comparable across study sites, because the scheduled analysis evaluated a higher dose.

The scheduled analysis assessed biodistribution one month after the P30-P35 administration of 8.8 × 10^11^ vg/mouse (*n* = 6–10 mice per TSHA-102-treated group; Figure S6). Viral genomes were detected throughout the central nervous system for female WT, male WT, and male KO mice treated with TSHA-102. These results confirm that IT injections were internally consistent.

The end-of-life analysis assessed biodistribution for the three lowest doses tested across the P7–P28 treatment ages (Figure S7A–F, Table S4). The mean viral genome copy numbers were a magnitude lower than those observed for the scheduled analysis. Many factors may have contributed to the lower biodistribution levels: lower doses, cell turnover, and/or variability in injections or tissue collection across studies. The results for the end-of-life analysis confirmed that injections were internally consistent among the JAX mice selected for analysis. Although not directly comparable, a similar fold difference in biodistribution for brain versus liver was observed for samples from both study sites. The CNS biodistribution profile was similar across treatment ages (Figure S7A–C). The heart and/or liver biodistribution increased with age (*n* = 3 TSHA-102-treated KO mice/group, Figure S7A–C) and dose (*n* = 3 TSHA-102-treated KO mice/group, Figure S7D–F).

### 3.13. MiniMECP2 Gene Expression Mirrored Biodistribution in KO Mice

Scheduled and end-of-life tissue samples were collected for gene expression analyses (Figures S8 and S9). End-of-life expression should be interpreted cautiously, because the regulatory activity of miRARE across development has not been clearly established. Both scheduled and end-of-life gene expression analyses should be interpreted cautiously, because the robust regulation mediated by miRARE can yield expression values near the lower quantification limit, creating datasets that contain both quantifiable (>LOQ) and non-quantifiable data points (<LOQ). Because of this blending of data types, we did not conduct a statistical analysis and rather only noted qualitative observations.

The scheduled gene expression in Figure S8 is a graphical representation of the tabulated data from NBS and includes both quantifiable data and non-quantifiable data (for which NBS assigned a threshold value; see raw data in Supplementary File S2). One fourth of the data points for TSHA-102-treated mice were BLOQ, and most of those were for the sciatic nerve (SN). Complete uncensored data were similar across genders and genotypes. The similar gene expression levels for WT and KO spinal cord mirror the similar protein expression levels previously published for myc-tagged TSHA-102 in WT and KO mice [8]; approximately 90% and 60% of the spinal cord and brain qPCR data points for TSHA-102-treated mice were quantifiable, respectively.

End-of-life gene expression data for lower doses yielded fewer quantifiable data points for the TSHA-102-treated mice (half of the data points were quantifiable). The lower expression levels mirror the lower biodistribution levels for the end-of-life study (compared to the scheduled study). Perhaps a clear way to visualize the whole data set, without assigning a fixed value to half of the data points, is to bin the data points according to their distribution above, within, and below the BLOQ range defined in the Materials and Methods (*n* = 3 TSHA-102-treated KO mice/group, Figure S9A). In the nervous tissue, detectable data points approached or exceeded the LOQ with an increasing dose, and detectable data points became less quantifiable with an increasing treatment age. For simplicity, Figure S9B highlights comparisons in which all data points were above the LOQ. In general, patterns in the hepatic and cardiac gene expression mirrored the biodistribution data (Figures S7–S9). Approximately 10% of the brain and cerebellar end-of-life qPCR data points for the mice treated with TSHA-102 at P28 were quantifiable.

### 3.14. MiniMeCP2 Protein Is Detectable in the Spinal Cords of Older KO Mice

A scheduled analysis (perfusion at 3 weeks post-injection) was selected to provide the clearest interpretation of miniMeCP2 protein expression data. KO mice were treated at P38 (IT; 2.2 × 10^11^ or 8.8 × 10^11^ vg/mouse; *n* = 3–5 mice/group) and perfused 3 weeks later (Figure S10).

MiniMeCP2 protein expression was highest in the spinal cord; no or low miniMeCP2 protein expression was observed across the brain (Figure S10A). Detectable protein was mostly observed in the pons and medulla (8.8 × 10^11^ vg/mouse). No expression was observed in the cerebellum and hippocampus. The undetectable or low protein expression in the brain (Figure S10) is consistent with the qPCR data herein revealing mini*MECP2* mRNA expression levels near the limit of quantification (Figure S9).

Sagittal tile scans (Figure S10B,C) show detectable miniMeCP2(+) cells in the upper spinal cord. These cells are an internal control validating the application of antibodies to sagittal brain sections, while highlighting the absence of detectable expression in the medulla at the low magnification used for tile scanning (signal is detectable in the medulla at higher magnification; Figure S10A,D,E).

Figure S10F–I shows cerebellar miniMeCP2 immunolabeling for a KO negative control (no primary antibody), a *Mecp2*^+/*y*^ positive control, and selected mice treated with TSHA-102 (2.2 × 10^11^ and 8.8 × 10^11^ vg/mouse). Even among mice with relatively high miniMeCP2 expression levels in the spinal cord, no protein was observed in the cerebellum. Importantly, the qPCR data herein show that the TSHA-102 vector reached the cerebellum after IT injection, and mini*MECP2* mRNA was expressed in the cerebellum (Figures S7 and S9).

At the cellular level, mice with the highest percentage of miniMeCP2(+) spinal cord cells showed a high expression in some of the positive cells, with miniMeCP2 localized to the nuclei, cytoplasm, and neuronal extensions (Figure S10J,K). Other cells showed the anticipated nuclear punctate localization of miniMeCP2 [9]. To be clear, these images (Figure S10B–K) were selected from mice with the highest expression levels in their respective treatment groups, specifically to emphasize the contrasting robust regulation that appears to occur in specific CNS regions.

### 3.15. TSHA-102 Is Well-Tolerated in Female Mice

Myc-tagged TSHA-102 has already been shown to be well-tolerated in male WT mice [8]. To further evaluate safety in alignment with a recently published female study [11], TSHA-102 was administered to adult female WT and *Mecp2*^−/+^ mice. Intrathecal TSHA-102 had no significant deleterious effect on survival versus vehicle-treated female mice during a 47-week study (*p* > 0.05, *n* per *Mecp2*^−/+^ group is 17–19 mice; 1–2 censored mice per vehicle- and TSHA-102-treated *Mecp2*^−/+^ group; Figure S11).

## 4. Discussion

### 4.1. The Large-Scale Efficacy Study Used Complementary Tests to Better Elucidate Phenotypes

The efficacy study conducted at JAX helped to enable the initiation of clinical testing for TSHA-102, an AAV9/mini*MECP2-*miRARE vector (clinical trial identifier number NCT05606614) [8,18]. In all, 14 TSHA-102 treatment paradigms were evaluated in KO mice at JAX, with treatment ages spanning from P2 to P28 and doses spanning from 8.8 × 10^10^ to 8.8 × 10^11^ vg/mouse (human equivalent doses of 2.9 × 10^14^–2.9 × 10^15^ vg/participant) [30]. To our knowledge, no other *MECP2* gene therapy study has evaluated efficacy in KO mice dosed at P7 and P14, the intermediate treatment ages evaluated herein. P7 in rodents approximately translates to late gestation in humans [31]. Many *MECP2* gene therapy studies published thus far have capped treatment at 4–5 weeks of age [6,7,8,9,10]. Likewise, the oldest treatment age in the efficacy study herein was capped at P28.

An expanded scope of tests was prioritized for older treatment ages (P7–P28) with the expectation that any observed efficacy would hold greater translational value. The additional tests permitted a granular quantification of phenotypes—particularly respiration—that would otherwise be coarsely assessed by the conventional severity scoring system (Bird score) for RTT mice [22]. One relative advantage of the aggregate scoring system is that it generates limb/motor data for all KO mice, whether they are spontaneously ambulatory or not. The narrow window of time between P28 and the median survival of vehicle-treated KO mice limits the number of tests that can be scheduled.

### 4.2. TSHA-102 Improves Survival, Weight, and Respiration across Treatment Ages

In general, each readout in the efficacy study showed that TSHA-102 was effective across multiple but not all treatment paradigms (dose, age, and route combinations). The neonatal administration of TSHA-102 provided similar types of benefits (improved survival, weight, and aggregate behavior) to those published for unregulated *MECP2* and mini*MECP2* gene therapies administered at similar doses to KO mice [6,7,12,15]. Neonatal administration also delayed the onset age for severe clasping and severely abnormal gait in KO mice (Figure 1).

TSHA-102 improved weight, respiration, and the median survival of KO mice treated IT at P7, P14, and P28 (Figure 2, Figure 3 and Figure 4, Table 1). One of the higher doses (4.4 × 10^11^ vg/mouse) frequently demonstrated efficacy across many treatment paradigms for many of the readouts (Figure 3, Figure 4 and Figure 6 and Table 1). The dose-matched (8.8 × 10^10^ vg/mouse) survival extension observed after older treatment ages was attenuated compared to that observed after P2 administration (P2 administration also uses a different route and volume; Figure 1).

To date, no AAV9/*MECP2* gene therapy has achieved WT-like survival in KO mice treated at 4–5 weeks of age [6,7,8,9,10,12,13,14,15,16]. Nonetheless, one may hypothesize that the observed survival efficacy may indirectly signal other improvements (e.g., respiratory well-being) that—if translated to patients—would improve quality of life and perhaps decrease the risk of sudden death for a subset of patients [23]. The observed respiratory efficacy is especially important, as most RTT patients experience breathing abnormalities at some point in their life [23]. Importantly, TSHA-102 achieved respiratory efficacy in KO mice without compromising survival in *Mecp2*^−/+^ mice (Figure S11). Safety in female RTT mice was a priority in light of previously published lethality in *Mecp2*^−/+^ mice after treatment with an alternative vector [10,11].

Regarding survival efficacy, it is important to note that the methods for accounting for veterinarian-requested euthanasia (unrelated to weight loss) are slightly different from those that we have previously published [8]. We have previously indicated veterinarian-requested euthanasia with symbols in our survival plots [8]. This approach communicates endpoints transparently while inviting important discussion. For example, if a veterinarian were to amputate a lesioned tail, could the mouse have survived longer without euthanasia? If so, it may be fair to censor (not exclude) data for the mouse. In contrast to our published methods, JAX censored the data for veterinarian-requested euthanasia. Both approaches are acknowledged in the Kaplan–Meier guidelines [32,33], and—conceptually—both approaches may sometimes potentially bias statistical outcomes. Ultimately, sound approaches for strengthening the survival conclusions include the use of an additional study arm (i.e., +CsA) and orthogonal efficacy readouts.

Other behavioral readouts (TreadScan, Bird scores, extracted sub-scores, and a portion of the respiratory data) indicated either no efficacy or efficacy that was limited in scope (Figure 5, Figure 6 and Figure S4; Table 2 and Table S2). These behavioral readouts warrant a thoughtful review of technical limitations. Important considerations for RTT mouse testing—unrelated to *MECP2* gene therapies—include: (1) the inability to assess gait for some non-ambulatory control mice via treadmill (Figure S5); the limited sensitivity of observer-generated scoring systems (Table 2 and Figure 5); interference from secondary interventions (Table S1 and Figure S3); the ability to consistently detect a baseline phenotype for vehicle-treated control mice (Figure S5); and the ability to maintain viability long enough to detect severe phenotypes in sick mice (Figure 5 and Table S2). Perhaps one of the most important technical considerations for the field of *MECP2* gene therapy is the age-dependent efficacy illustrated by 10 years of *MECP2* gene therapy publications [6,7,8,10,12,14,15,16,17]. The two extreme paradigms tested herein (P2 ICV and P28 IT administration) yielded aggregate severity scores consistent with many publications featuring AAV9 vectors (i.e., better efficacy and little or no efficacy, respectively; Figure 1 and Figure S1; Table 2) [6,7,9]. Ultimately, a strength of this study is the presentation of TSHA-102 efficacy across several (if not all) readouts, doses, and treatment ages, despite the limitations listed above.

### 4.3. The CsA Study Arm Builds upon a Valuable Research Contribution from Our Peers

Preclinical studies have relied almost exclusively on KO mice, partly because the female mouse model is cost-prohibitive and technically challenging to work with due to its delayed onset of symptoms, inherent phenotypic variability, and mild symptoms (versus KO) [6,7,8,9,10,12,13,14,34]. Unfortunately, KO mice can experience an immune response to exogenous MeCP2, and that immune response may shorten survival [13]. In agreement with previously published results, the CsA regimen described herein decreased the occurrence of tail lesions warranting euthanasia in virus-treated KO mice [13]. Notably, most virus-treated WT and *Mecp2*^−/+^ mice evaluated herein never developed tail lesions.

The study herein also presents a cautionary tale. On one hand, the absence of immunosuppression can decrease KO survival [13]. On the other hand, daily immunosuppression may improve KO respiration (Figure S3). The mechanistic explanation for the latter observation is unclear. For example, the improved respiratory efficacy for +CsA vehicle-treated mice (versus -CsA vehicle-treated mice) may be mediated by the CsA peptide itself or by the daily handling of the mice. The latter would align with Pitcher et al.’s observation that frequent handling can improve KO data (2013) [35]. Solutions for the observed interference by CsA may include evaluating an alternative immunosuppression regimen or testing a different mouse model of RTT. Ultimately, the study design described herein permits clear conclusions: even with tightly regulated expression, a single dose of TSHA-102 can improve survival and respiration in KO mice.

### 4.4. Further Exploration of miRARE-Mediated Expression Regulation Is Warranted

The mechanistic underpinnings of miRARE-mediated expression have yet to be fully elucidated. Published data showed that miRARE regulates expression in multiple brain regions and the spinal cord in both WT and KO mice (AAV9/mini*MECP2-myc-*miRARE versus AAV9/mini*MECP2-myc*) [8]. Evidence of genotype-dependent protein expression has, thus far, been limited to the pons and midbrain after the P28-P35 IT administration of myc-tagged TSHA-102 in male WT and KO mice [8]. Indeed, we have previously discussed the importance of validating these genotype-dependent expression observations via an orthogonal approach (e.g., a mosaic fluorescence mouse model) [8]. Thus, the near-zero expression observed in the midbrain and low expression observed in the pons of KO mice were particularly surprising and may warrant careful planning before initiating expression studies in mosaic mice (P38 IT administration; Figure S10; compare to the ~30–35% of miniMeCP2-myc(+) cells previously observed in KO pons and midbrain after treating mice aged 4–5 weeks [8]). Conceptually, both published and new observations may be reconcilable. One should also recognize that low and potentially therapeutic levels of miniMeCP2 might be expressed, but not reliably detected due to method sensitivities or other variables—the therapeutic benefits seen in treated mice suggest this. Finally, the observed—albeit low—expression in the medulla and pons may explain why respiratory efficacy was achieved, despite the globally robust regulation mediated by miRARE in the brain.

Future exploration of miRARE-regulated protein expression should address multiple variables, including dose, treatment age, assessment ages, the tissue-specific endogenous miRNA profile (across developmental stages), and gender. The potential overcorrection observed for some (not all) TreadScan parameters in vector-treated mice suggests that the regulation of miniMeCP2 may need to be further refined (Figure 6). Alternatively, the observed overcorrection may be an artifact of small group size for ambulatory vector-treated mice (4.4 × 10^11^ vg/mouse). Last, a head-to-head comparison of mini*MECP2*-miRARE vectors (with and without the myc tag; with the same fixation, primary antibody, and antigen retrieval conditions) may reveal the effect of the transgene design, if any, on miRARE function. Smaller mRNAs are known to be less resistant to RNA interference [36]. If this holds true for miniMeCP2, then one may hypothesize that miRARE-mediated inhibition of miniMeCP2 may vary more sharply between tissues that do and do not express the cognate miRNAs. An alternative explanation—the destabilization of protein expression after myc tag removal—would more likely impact expression levels uniformly across tissues, regardless of their miRNA profiles. Reassuringly, the spinal cord protein expression described herein is similar to what we have previously published for the myc-tagged vector [8]. The absence of hippocampal protein expression is consistent with the low hippocampal protein expression levels previously published for myc-tagged TSHA-102 [8]. Although we have not previously published cerebellar protein expression data in the peer-reviewed literature, the observed lack of cerebellar protein expression is consistent with past anecdotal observations (TSHA-102 in KO mouse) and non-peer-reviewed data in the patent literature (PHP.B/mini*MECP2*-*myc*-miRARE in WT mice; Filing # WO2020047234) [37].

The lack of cerebellar protein expression is not entirely surprising, given the relative abundance of endogenous miR-9-5p in the cerebellum versus other brain regions in both male and female humans across developmental stages (see supplementary data published by Ziats and Rennert) [38]. Indeed, miRARE may potentially have a second binding site for miR-9-5p that may enhance regulation in the cerebellum. One seed match for miR-9-5p was designed intentionally; the second seed match (which may or may not function efficiently) was inadvertently created through the juxtaposition of two flanking sequences for the miR-26-5p and miR-23-3p binding sites in miRARE [8]. Further miRARE design modification, if any, should proceed with either untagged mini*MECP2* or both tagged and untagged genes in parallel.

## 5. Conclusions

The TSHA-102 data presented herein provide pivotal evidence for treatment benefits to support the regulatory approval of the initiation of a human clinical trial (clinical trial identifier number NCT05606614) [18]. Most importantly, TSHA-102 demonstrated efficacy across multiple treatment ages and doses in KO mice. The respiratory and survival data present the strongest evidence for efficacy. The protein expression levels were low in the pons and medulla, as well as elsewhere in the brain; the protein expression levels were higher in the spinal cord. Follow-up studies evaluating myc-tagged and human-ready TSHA-102 vectors in parallel will help to resolve questions about protein detection sensitivity and the consequences of myc tag removal. Further pre-clinical studies will help researchers to better understand the scope and detailed mechanism of miRARE-mediated regulation. The safety and efficacy of TSHA-102 are favorable and supportive of ongoing clinical efforts.

## Figures and Tables

**Table 1:** TSHA-102 improves median survival after administration in older KO mice.

Genotype, Treatment	Median Survival (Weeks) after the Indicated Treatment Ages		
	P7	P14	P28
WT	>15	>15	>15
KO	8.4	9.7	9.55
KO, 8.8 × 10^10^ vg/mouse	10 **	12 *	11
KO, 2.2 × 10^11^ vg/mouse	10 **	12.6 **	14 *
KO, 4.4 × 10^11^ vg/mouse	10.1 *	11.9 **	12 *
KO, 8.8 × 10^11^ vg/mouse	NE	NE	15 **
KO, CsA	NE	9.9	9.9
KO, CsA + 8.8 × 10^10^ vg/mouse	NE	NE	NE
KO, CsA + 2.2 × 10^11^ vg/mouse	NE	NE	NE
KO, CsA + 4.4 × 10^11^ vg/mouse	NE	12.1 **	11.6
KO, CsA + 8.8 × 10^11^ vg/mouse	NE	NE	15.5 **

Key results are bolded. The number of KO mice assigned per treatment age is: (P7) 11–12; (P14) 12; (P14 + CsA) 12–13; (P28) 12; and (P28 + CsA) 12. The number of WT mice assigned per treatment age is 12. Some KO mice were censored due to severe tail lesions or other events (see Materials and Methods). The remaining *n* per group is: (P7) 7–12; (P14) 10–12; (P14 + CsA) 6–11; (P28) 8–12; and (P28 + CsA) 11–12. Among KO mice treated at P28 with 8.8 × 10^11^ vg/mouse (without CsA), 4 developed lesions warranting euthanasia and were censored; none of the dose- and age-matched immunosuppressed KO mice developed these lesions. The highest dose (8.8 × 10^11^ vg/mouse) could not be administered at P7 and P14 due to injection volume limits for those ages. NE: Not enrolled. * *p* ≤ 0.05 and ** *p* ≤ 0.01, versus the appropriate vehicle-treated control (±CsA).

**Table 2:** Treatment effect on KO aggregate severity scores after TSHA-102 administration at P7, P14, or P28.

Treatment	Weeks with Significant Post-Hoc Differences/Total Weeks Analyzed		
	P7	P14	P28
8.8 × 10^10^ vg/mouse	2/10	0/8	0/9
2.2 × 10^11^ vg/mouse	2/10	0/9	0/9
4.4 × 10^11^ vg/mouse	2/10	2/8	0/9
8.8 × 10^11^ vg/mouse	NE	NE	0/9
CsA + 8.8 × 10^10^ vg/mouse	NE	NE	NE
CsA + 2.2 × 10^11^ vg/mouse	NE	NE	NE
CsA + 4.4 × 10^11^ vg/mouse	NE	0/9	0/9
CsA + 8.8 × 10^11^ vg/mouse	NE	NE	2/9

The number of weeks in which a statistical post-hoc difference (*p* ≤ 0.05 after a two-way ANOVA accounting for all doses in the respective treatment age and study arm) was observed is shown. Comparisons are made to the vehicle-treated KO control group for the respective arm (−CsA or + CsA). NE: Not enrolled. The *n* per treatment age is: (P7) 10–12; (P14) 10–12; (P14 + CsA) 6–11; (P28), 11–12; and (P28 + CsA) 12.

## Data Availability

Data are either provided herein or are available upon request. Medical, legal, and regulatory interests for multiple parties must be considered prior to any release of requested data.

## Supplementary Materials

The following supporting information can be downloaded at: https://www.mdpi.com/article/10.3390/genes15010031/s1, Figure S1: A summary of selected AAV9/*MECP2* gene therapies for the preclinical treatment of RTT in male KO mice; Figure S2: Certificates of Testing; Table S1: Respiration summary for immunosuppressed KO mice after gene therapy administration; Figure S3: Daily injections of CsA may affect respiration in vehicle-treated KO controls; Figure S4: A brief dip in Bird scores was observed at 6 weeks of age after P7 administration; Table S2: Age of onset comparisons among KO mice after gene therapy administration at P7, P14, or P28 (−CsA); Table S3: Age of onset comparisons among +CsA KO mice after gene therapy administration; Figure S5: TreadScan data for mice treated at P7, P14 (+CsA), P28, and P28 (+CsA); Figure S6: Biodistribution of TSHA-102 one month after injection; Figure S7: End-of-life biodistribution of TSHA-102 administered at JAX; Table S4. Survival range of mice selected for end-of-life qPCR analyses; Figure S8: Gene expression analyses one month after injection for TSHA-102 administered at UTSWMC; Figure S9: End-of-life gene expression of TSHA-102 administered at JAX; Figure S10: Expression of miniMeCP2 protein is observed primarily in the spinal cord after P38 administration in KO mice. Figure S11: TSHA-102 is well-tolerated in a survival safety assessment in female WT and *Mecp2^−/+^* mice.
