# Supplementary material for: The Efficacy of a Human-Ready miniMECP2 Gene Therapy in a Pre-Clinical Model of Rett Syndrome
Source: Genes (Basel). 2023 Dec 24;15(1):31. doi: 10.3390/genes15010031 (PMC10815157; doi:10.3390/genes15010031)
Supplement: Supplementary file 1 [file genes-15-00031-s001.zip › 12.23.23 Supplementary Figures .pdf]

SUPPLEMENTAL INFORMATION

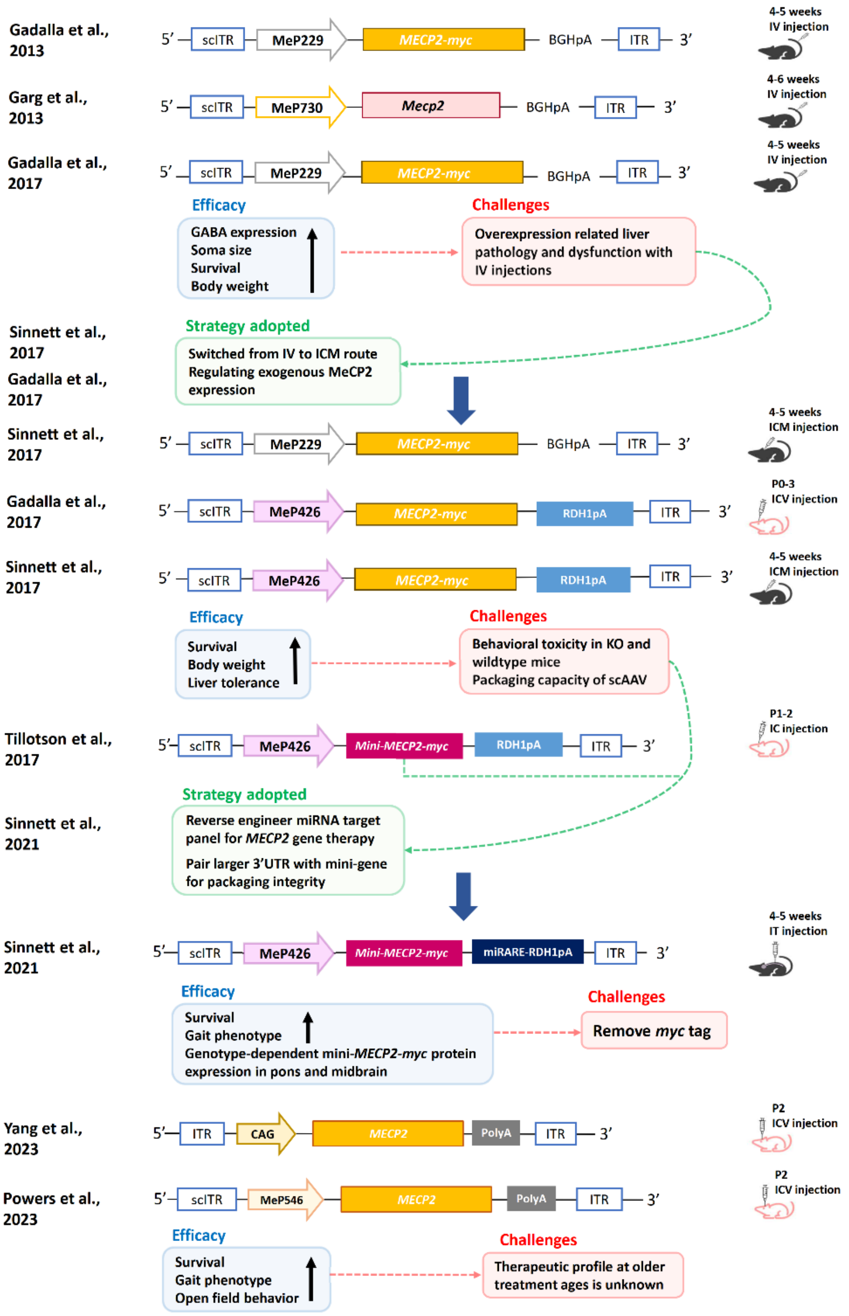

Figure S1. A summary of selected AAV9/*MECP2* gene therapies for the preclinical treatment of RTT in male KO mice. Many *MECP2* gene therapy publications from the past decade are referenced herein [6-9,12,14-17]. Published information regarding the AAV9/MeP229-*MECP2-myc* vector after neonatal administration is limited to an anecdote in the original publication's discussion [6]. Not illustrated herein: single-stranded AAV9/CBA-*MECP2-myc*-SV40pA,

which was abandoned [6]; and scAAV9/MeP223-(codon-optimized)*MECP2* tested in male and female mouse models of RTT [10,11]. BGHpA, bovine growth hormone poly(A); CAG, cytomegalovirus early enhancer/chicken β-actin; IC, intracranial; ICM, intracisterna magna; ICV, intracerebroventricular; IT, intrathecal; ITR, inverted terminal repeats; IV, intravenous; MeP223-MeP730 are fragments of the endogenous *MECP2* promoter; mini-*MECP2*, mini*MECP2* gene is human-derived [12]; miRARE, miRNA-responsive auto-regulatory element [8]; RDH1pA, synthetic 3'UTR containing miRNA targets [7,9]; sc, self-complementary (sc); SV40pA, simian virus 40 poly(A).

Figure S2. Certificates of testing.

Catalent
CELL & GENE THERAPY

Certificate of Testing

**Product Name**	Client 114, 102 Molecule 2020-121, Non-GMP Drug Product
**LOT#**	114-0321-349
**Manufacturing Date**	14APR2021

Release Testing

Description	Tested By	Test Request Number	Test Result
Endotoxin	BioPark QC	TR#137847	0.0125 EU/mL
Bioburden	BioPark QC	TR#137846	TAMC < 1 CFU/ mL<br>TYMC < 1 CFU/ mL
AAV Titer (ddPCR)	BioPark AD	Analysis ID #21-3927	$8.76x10^{13}$ vg/mL
Capsid Titer (ELISA)	BioPark AD	Analysis ID #21-3928	$2.48x10^{14}$ cp/mL
Residual Host Cell DNA with SAN Treatment	BioPark AD	Analysis ID #21-4065	8901.633 ng/mL<br>1016 ng/$1x10^{13}$ vg copy
Residual Host Cell DNA without SAN Treatment	BioPark AD	Analysis ID #21-4065	9563.000 ng/mL<br>1092 ng/$1x10^{13}$ vg copy
Residual E1A without SAN Treatment	BioPark AD	Analysis ID #21-3932	$1.77x10^{7}$ cp/mL<br>$2.02x10^{6}$ E1A/$1x10^{13}$ vg copy
Residual Host Cell Protein (HEK293)	BioPark AD	Analysis ID #21-3935	< 4 ng/mL
Residual AAV9 Ligand	BioPark AD	Analysis ID #21-3939	95.29 ng/mL
Residual Benzonase	BioPark AD	Analysis ID #21-3938	< 1.55 ng/mL
Residual Plasmid DNA (Kanamycin) without SAN Treatment	BioPark AD	Analysis ID #21-3931	$6.09x10^{12}$ copies/mL<br>$6.95x10^{11}$ Kan/ $1x10^{13}$ vg copy
Residual Helper DNA (pALD-X80) without SAN Treatment	BioPark AD	Analysis ID #21-3930	$3.99x10^{10}$ copies/mL<br>$4.55x10^{9}$ Helper/ $1x10^{13}$ vg copy

Page 1 of 3

Catalent
CELL & GENE THERAPY

CE-SDS (A220)	BioPark AD	Analysis ID #21-3936	91.8% VP1, VP2, and VP3
SEC-UPLC (A220)	BioPark AD	Analysis ID #21-3937	98.2% Monomer
Appearance	BioPark QC	TR#: 137848	Colorless, opalescent between III and IV reference solutions, no visible particulates
Osmolality	BioPark QC	TR#: 137850	561 mOsm/kg H_2O
pH	BioPark QC	TR#: 137849	7.4
TCID50	BioPark AD	Analysis ID #21-3934	$2.00x10^{12}$ IU/mL<br>$2.28x10^{11}$ IU/$1x10^{13}$ vg copy
AUC	BioPark AD	Analysis ID #21-4149	32% Empty

Results reviewed for Accuracy and Completeness

AUC Sedimentation Profile:

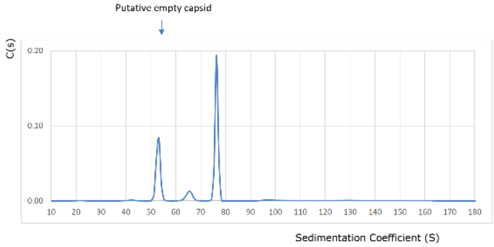

Page 2 of 3

Catalent
CELL & GENE THERAPY

Approvals

Nagendra Singh, Senior Scientist, Downstream Process Development at Catalent Gene Therapy	01 Jun 2021<br>Date
Tao Wang, Manager, Downstream Process Development at Catalent Gene Therapy	01JUN2021<br>Date

Page 3 of 3

UTSouthwestern
Medical Center

Translational Gene Therapy Core (TGTC)

Certificate of Analysis Form

**Batch ID:**	RS-14-20-001 and RS-14-20-002
**Product Name:**	pAAV9 / KanR-miniMecP2-miRARE
**ITR Plasmid Name:**	KanR-miniMecP2-miRARE
**Manufacturing Date:**	12JAN2021
**Manufacturing Scale:**	30L
**Final product volume:**	0.8mL
**Storage Conditions:**	≤-60˚C, Upon thawing, 4˚C

Test	Method	Results
Endotoxin	LAL	2.49 EU/mL
Capsid Identity	ELISA	n/a
Capsid Identity	Stunner	n/a
Titer-Vg	qPCR, target ITR	1.17E+14 Vg/mL
Titer-Vg	Stunner	n/a
Purity	Silver Stain	n/a

- COA is not provided for RS batch (research batch). All the results listed above are based on the mass balance and endotoxin record.

Released By Yang Yu

Table S1. Respiration summary for immunosuppressed KO mice after gene therapy administration

vg/mouse	Difference in Breathing Frequency (versus Vehicle + CsA)		
	**P7**	**P14**	**P28**
8.8×10^{10}	NE	NE	NE
2.2×10^{11}	NE	NE	NE
4.4×10^{11}	NE	NS	***$p \leq 0.05$[a] (Improvement)**
8.8×10^{11}	NE	NE	NS
**vg/mouse**	**Difference in Expiratory Time (versus Vehicle + CsA)**		
	**P7**	**P14**	**P28**
8.8×10^{10}	NE	NE	NE
2.2×10^{11}	NE	NE	NE
4.4×10^{11}	NE	NS	***$p \leq 0.05$[a] (Improvement)**
8.8×10^{11}	NE	NE	NS
**vg/mouse**	**Difference in Inspiratory Time (versus Vehicle + CsA)**		
	**P7**	**P14**	**P28**
8.8×10^{10}	NE	NE	NE
2.2×10^{11}	NE	NE	NE
4.4×10^{11}	NE	***$p \leq 0.05$ (Decrement)**	NS
8.8×10^{11}	NE	NE	NS
**vg/mouse**	**Difference in Apnea Duration (versus Vehicle + CsA)**		
	**P7**	**P14**	**P28**
8.8×10^{10}	NE	NE	NE
2.2×10^{11}	NE	NE	NE
4.4×10^{11}	NE	NS	NS
8.8×10^{11}	NE	NE	NS
**vg/mouse**	**Difference in Apnea Frequency (versus Vehicle + CsA)**		
	**P7**	**P14**	**P28**
8.8×10^{10}	NE	NE	NE
2.2×10^{11}	NE	NE	NE
4.4×10^{11}	NE	NS	NS
8.8×10^{11}	NE	NE	NS

Key results are bolded. Treatment ages (P7, P14, and P28) are listed at the top of each column. Comparisons were not made across treatment ages because the timespan between treatment age and testing varied across groups. A t-test compared the only 2 KO groups (vehicle versus 4.4 x 10^{11} vg/mouse) evaluated after P14 gene therapy administration (+CsA); a one-way ANOVA followed by post-hoc comparisons compared KO groups (vehicle versus 4.4. x 10^{11} or 8.8 x 10^{11} vg/mouse) evaluated after P28 gene therapy administration. With few exceptions, most of the respiratory readouts for groups enrolled above showed no difference between immunosuppressed vehicle-treated KO mice and immunosuppressed TSHA-102-treated mice. n = 5-11 KO mice/group (after P14 administration); n = 12 KO mice/group after P28 administration. NE, not enrolled; NS, not significant. Also, see results for the non-immunosuppressed arm of the study in **Figures 3-4**.

[a]Indicates consistent observation across non-immunosuppressed and immunosuppressed arms.

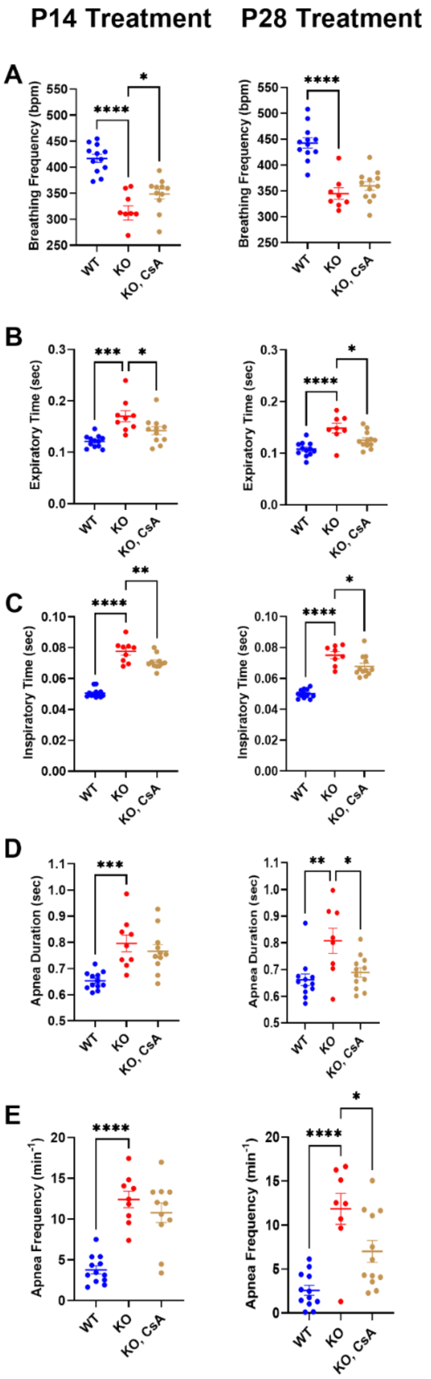

Figure S3. Daily injections of CsA may affect respiration in vehicle-treated KO controls. The CsA regimen (either the daily handling or the peptide itself) appeared to improve (**A**) breathing frequency, (**B**) expiratory time, (**C**) inspiratory time, (**D**) apnea duration, and (**E**) apnea frequency in KO mice treated with vehicle after P14 and/or P28 administration. Data are mean ± SEM. *n* per group: (P14) 9-12; (P28) 8 -12.

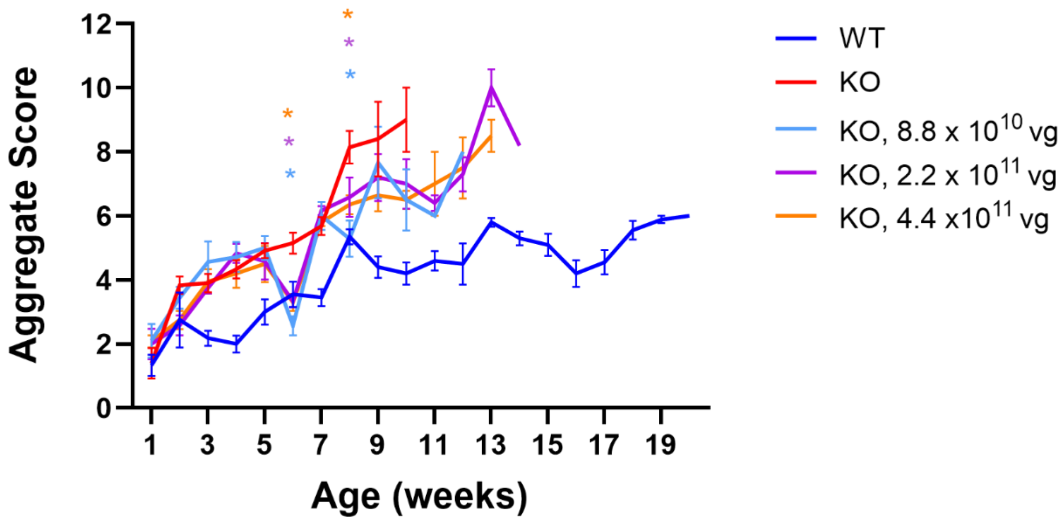

Figure S4. A brief dip in Bird scores was observed at 6 weeks of age after P7 administration. Although not notated, significant differences were observed between vehicle-treated KO and WT groups starting at 3 weeks of age. Data are mean ± SEM. The *n* per KO group is 10-12; WT *n* = 12.

Table S2. Age of onset comparisons among KO mice after gene therapy administration at P7, P14, or P28 (-CsA).

vg/mouse	Difference in Severe Breathing Onset (versus Vehicle)		
	**P7**	**P14**	**P28**
8.8 x 10^{10}	NS	****$p \leq 0.01$, n = 5 KO mice/group**	NS
2.2 x 10^{11}	NS	***$p \leq 0.05$, n = 5-6 KO mice/group**	NS
4.4 x 10^{11}	NS	***p ≤ 0.05, n = 3-5 KO mice/group**	NS
8.8 x 10^{11}	NE	NE	**No severe scores for TSHA-102**
**vg/mouse**	**Difference in Severe Clasping Onset (versus Vehicle)**		
8.8 x 10^{10}	NS	***$p \leq 0.05$, n = 6-7 KO mice/group**	NS
2.2 x 10^{11}	NS	***$p \leq 0.05$, n = 6-10 KO mice/group**	NS
4.4 x 10^{11}	NS	NS	NS
8.8 x 10^{11}	NE	NE	NS
**vg/mouse**	**Difference in Severe Gait Onset (versus Vehicle)**		
8.8 x 10^{10}	NS	NS	NS
2.2 x 10^{11}	NS	NS	NS
4.4 x 10^{11}	NS	NS	NS
8.8 x 10^{11}	NE	NE	****$p \leq 0.01$ n = 4-7 KO mice/group**
**vg/mouse**	**Difference in Severe Mobility Onset (versus Vehicle)**		
8.8 x 10^{10}	NS	NS	NS
2.2 x 10^{11}	**No severe scores for TSHA-102**	NS	NS
4.4 x 10^{11}	NS	NS	NS
8.8 x 10^{11}	NE	NE	NA
**vg/mouse**	**Difference in Severe Tremors Onset (versus Vehicle)**		
8.8 x 10^{10}	NS	NA	NS
2.2 x 10^{11}	NS	NA	NS
4.4 x 10^{11}	NS	NA	NS
8.8 x 10^{11}	NE	NE	NS
**vg/mouse**	**Difference in Severe Condition Onset (versus Vehicle)**		
8.8 x 10^{10}	NS	NS	NS
2.2 x 10^{11}	NS	NS	NS
4.4 x 10^{11}	NS	**No severe scores for TSHA-102 group**	NS
8.8 x 10^{11}	NE	NE	NS

Key results are bolded. Treatment ages (P7, P14, and P28) are listed at the top of each column. Each *n* per group listed above refers to the number of mice presenting severe scores in each group. All significant p values indicate

improvement after gene therapy. NA, non-applicable because there was only 1 data point for the vehicle-treated group (severe tremors) or the TSHA-102-treated group (severely abnormal mobility); NE, not enrolled; NS, not significant.

Table S3. Age of onset comparisons among +CsA KO mice after gene therapy administration

vg/mouse	Difference in Severe Breathing Onset (versus Vehicle + CsA)		
	**P7**	**P14**	**P28**
8.8 x 10^{10}	NE	NE	NE
2.2 x 10^{11}	NE	NE	NE
4.4 x 10^{11}	NE	**NS**[a]	**NS**[a]
8.8 x 10^{11}	NE	NE	NS
**vg/mouse**	**Difference in Severe Clasping Onset (versus Vehicle + CsA)**		
8.8 x 10^{10}	NE	NE	NE
2.2 x 10^{11}	NE	NE	NE
4.4 x 10^{11}	NE	**NS**[a]	**NS**[a]
8.8 x 10^{11}	NE	NE	**NS**[a]
**vg/mouse**	**Difference in Severe Gait Onset (versus Vehicle + CsA)**		
8.8 x 10^{10}	NE	NE	NE
2.2 x 10^{11}	NE	NE	NE
4.4 x 10^{11}	NE	**NS**[a]	***$p \leq 0.05$; n = 8 affected KO mice/group**
8.8 x 10^{11}	NE	NE	***$p \leq 0.05$[a]; n = 8-11 affected KO mice/group**
**vg/mouse**	**Difference in Severe Tremor Onset (versus Vehicle + CsA)**		
8.8 x 10^{10}	NE	NE	NE
2.2 x 10^{11}	NE	NE	NE
4.4 x 10^{11}	NE	**NA**[a]	**NS**[a]
8.8 x 10^{11}	NE	NE	***$p \leq 0.05$; n = 4-5 affected KO mice/group**
**vg/mouse**	**Difference in Severe Mobility Onset (versus Vehicle + CsA)**		
8.8 x 10^{10}	NE	NE	NE
2.2 x 10^{11}	NE	NE	NE
4.4 x 10^{11}	NE	***$p \leq 0.05$; n = 2-3 affected KO mice/group**	**NS**[a]
8.8 x 10^{11}	NE	NE	NS
**vg/mouse**	**Difference in Severe Condition Onset (versus Vehicle + CsA)**		
8.8 x 10^{10}	NE	NE	NE
2.2 x 10^{11}	NE	NE	NE
4.4 x 10^{11}	NE	NA	**NS**[a]
8.8 x 10^{11}	NE	NE	**NS**[a]

Significant differences indicate improvement. NA, non-applicable because 0-1 of the vehicle-treated control mice scored a severe phenotype. NE, not enrolled; NS, not significant. Also, see the results for the non-immunosuppressed arm of the study in **Figure 5**.

[a]Indicates consistent observation across non-immunosuppressed and immunosuppressed arms.

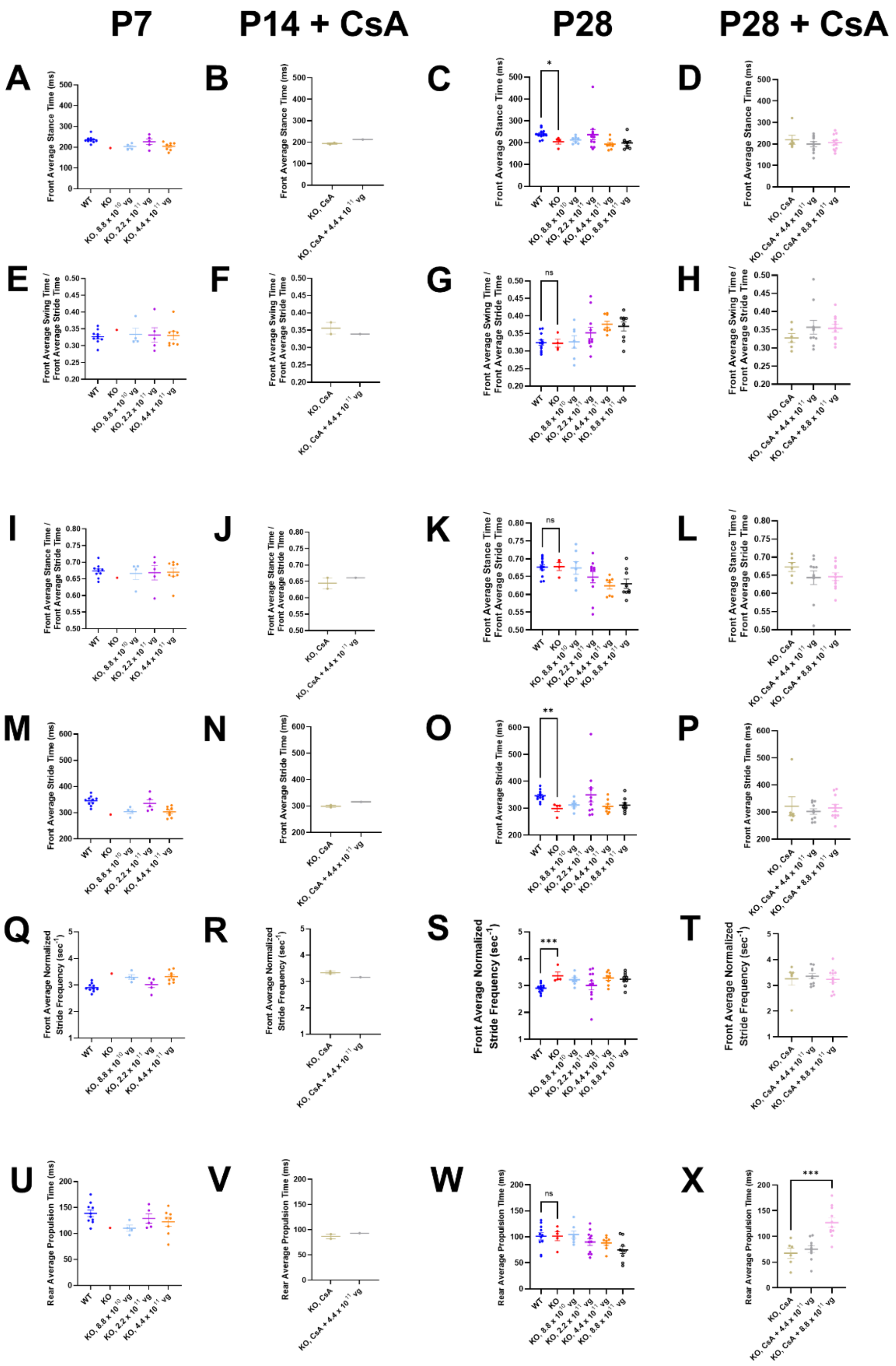

Figure S5. TreadScan data for mice treated at P7, P14 (+CsA), P28, and P28 (+CsA). Individual host data are provided to illustrate key technical details for the control groups. For example, some controls groups had few ambulatory mice (administration at P7 (-CsA) and P14 (+CsA)). Statistics are not presented for **A-B**, **E-F**, **I-J**, **M-N**, **Q-R**, and **U-V**. (**C, G, K, O, S, W**) Statistics are shown for the control groups to highlight readouts with a significant assay window between vehicle-treated controls. Although not notated for simplicity, there was no significant difference between vehicle and TSHA-102-treated mice after P28 administration. Data are means ± SEMs. ns, not significant. *n* per group: (P7) 1-10; (P14 + CsA) 1-2; (P28) 4-12; and (P28 + CsA) 6-11.

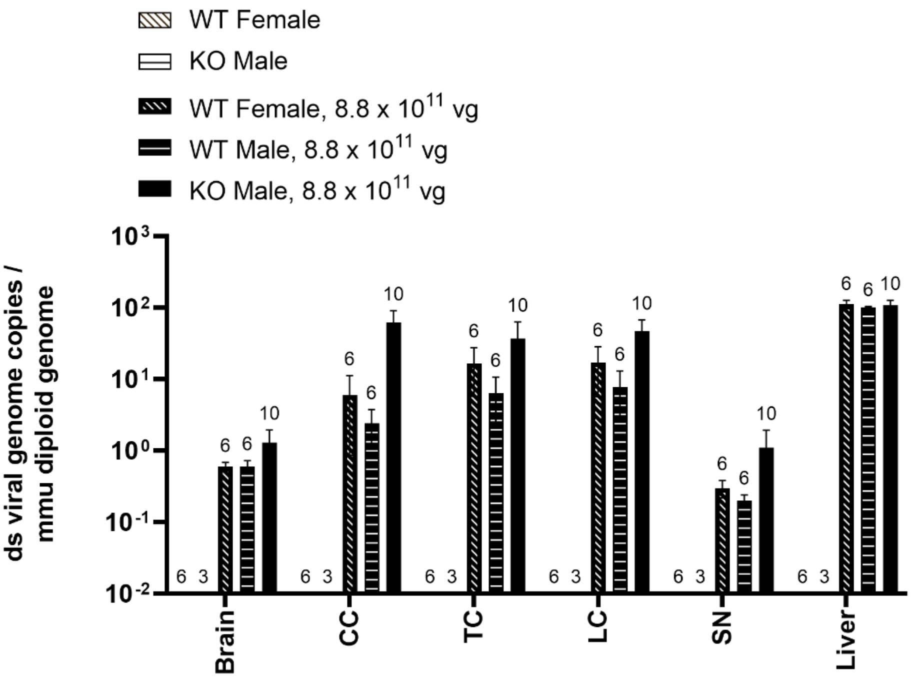

Figure S6. Biodistribution of TSHA-102 one month after injection. Tissues were collected one month after P30-P35 administration at UTSWMC. All data points for TSHA-102-treated mice were quantifiable by qPCR. *n* per group is listed above each bar. Data are mean ± SEM. CC, cervical spinal cord; LC, lumbar cord; mmu, Mus musculus; SN, sciatic nerve; TC, thoracic spinal cord.

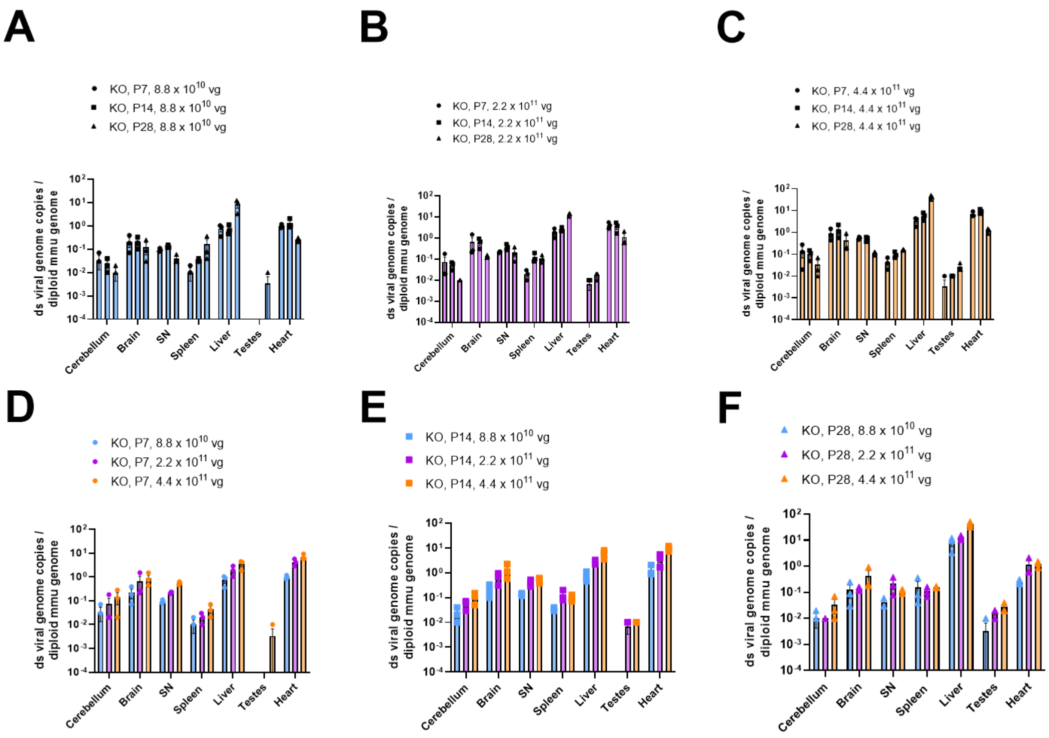

Figure S7. End-of-life biodistribution of TSHA-102 administered at JAX. Age-dependent biodistribution after administration of (**A**) 8.8 x 10^{10}; (**B**) 2.2 x 10^{11}; and (**C**) 4.4 x 10^{11} vg/mouse. (**D-F**) Dose-dependent biodistribution after treatment at (**D**) P7, (**E**) P14, (**F**) and P28. The CNS biodistribution was similar for each treatment age. A reasonable range in the dose-dependent increase in biodistribution is observed. (**A-F**) All data points for TSHA-102-treated mice were quantifiable by qPCR. *n* per group graphed is 3. All vehicle data was 0 vg per diploid mouse (mmu) genome. For simplicity, only TSHA-102 data is graphed. Missing bars for testes indicate an average value of 0.00 for *n* = 3 mice/group. The same legend applies for **A-C**; and for **D-F**. Data are mean ± SEM. SN, sciatic nerve.

Table S4. Survival range of mice selected for end-of-life qPCR analyses

Genotype, Treatment	Survival range (weeks)		
	P7	P14	P28
WT	20.9-21.0	21.9	NA
KO	8.4-10.4	7.0-9.9	NA
KO, 8.8 x 10^{10} vg/mouse	9.1-12.3	7.4-12.9	9.3-16.0
KO, 2.2 x 10^{11} vg/mouse	13.1-13.7	6.1-13.1	14.0-16.7
KO, 4.4 x 10^{11} vg/mouse	8.7-12.6	12.4-18.9	12.1-16.9
KO, 8.8 x 10^{11} vg/mouse	NE	NE	NA
KO, CsA	NA	NA	NA
KO, CsA + 8.8 x 10^{10} vg/mouse	NE	NE	NE
KO, CsA + 2.2 x 10^{11} vg/mouse	NE	NE	NE
KO, CsA + 4.4 x 10^{11} vg/mouse	NE	NA	NA
KO, CsA + 8.8 x 10^{11} vg/mouse	NE	NE	NA

NA, non-applicable because the enrolled groups were not evaluated for qPCR; NE, not enrolled. Mice found dead were not used for qPCR analyses. The same mice were used for biodistribution and gene expression analyses. *n* = 2 mice per enrolled vehicle-treated group; *n* = 3 mice per each TSHA-102-treated group evaluated.

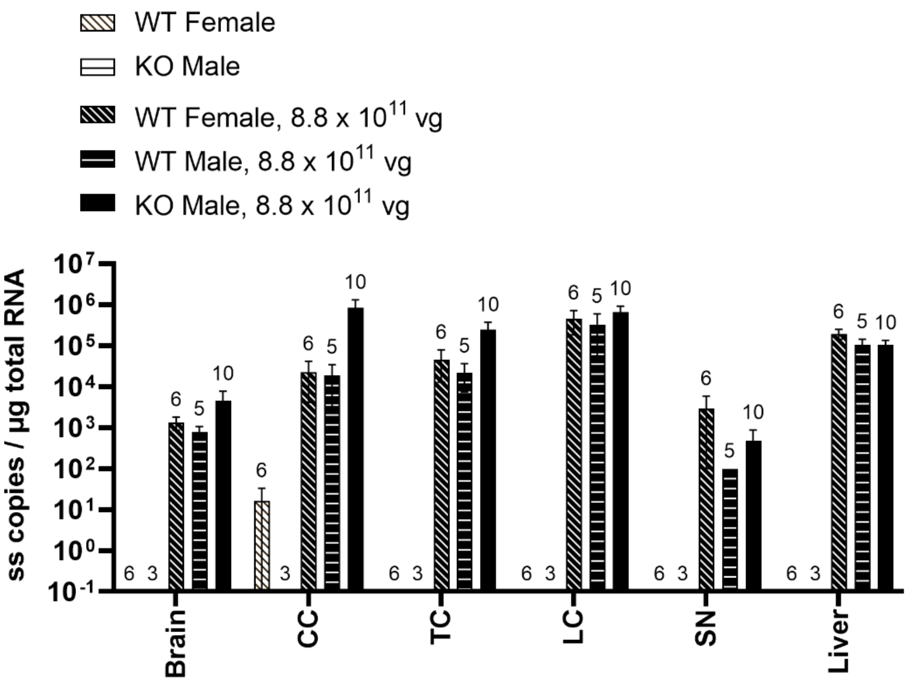

Figure S8. Gene expression analyses one month after injection for TSHA-102 administered at UTSWMC. Tissue was collected one month after treatment at P30-P35. *n* per group is listed above each bar. Approximately 75% of the data points for TSHA-102-treated mice were quantifiable. See also Supplementary File 2, which provides raw data identifying quantifiable and BLOQ data points. Data are mean ± SEM. CC, cervical spinal cord; LC, lumbar cord; SN, sciatic nerve; TC, thoracic spinal cord.

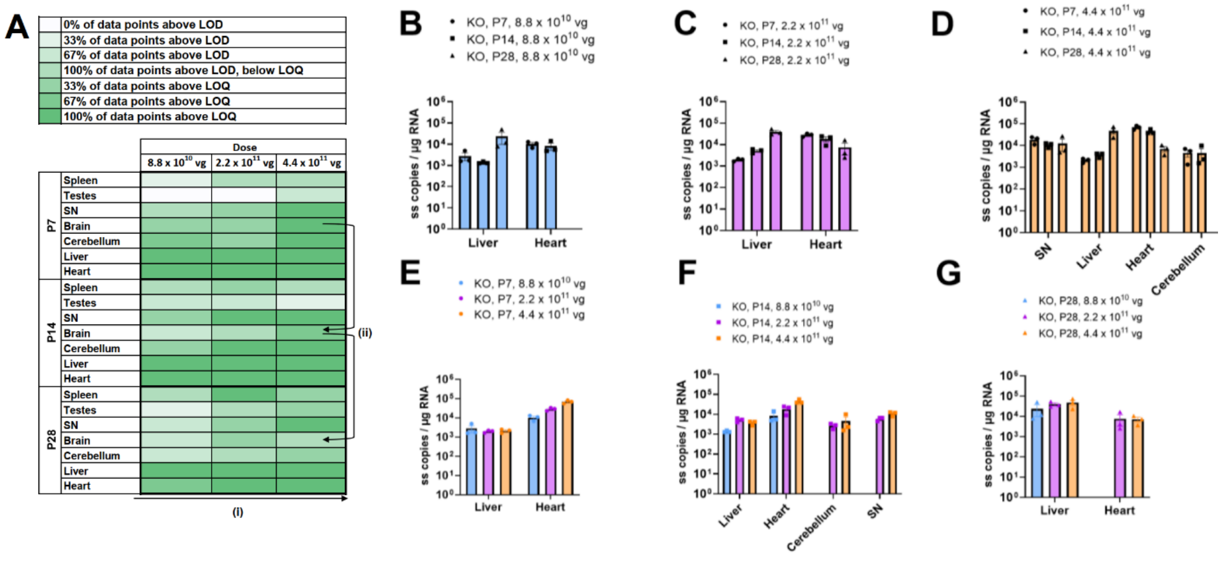

Figure S9. End-of-life gene expression of TSHA-102 administered at JAX. (**A**) Binning of expression data as non-detectable, detectable but not quantifiable, or quantifiable. *n* per group shown is 3. (**i**) In nervous tissue, detectable data points approached or exceeded the limit of quantification (LOQ) with increasing dose. (**ii**) In nervous tissue, detectable data points become less quantifiable with increasing treatment age. Arrows point to brain data across treatment ages. Quantifiable age-dependent gene expression after administration of (**B**) 8.8 x 10^{10}; (**C**) 2.2 x 10^{11}; and (**D**) 4.4 x 10^{11} vg/mouse; quantifiable dose-dependent gene expression after treatment at (**E**) P7, (**F**) P14, (**G**) and P28. (**B-G**) *n* per group shown is 3. The graphs show only groups in which all 3 data points are quantifiable. Thus, the end-of-life and scheduled biodistribution graphs are not directly comparable. Data are mean ± SEM. Patterns in gene expression resemble those in end-of-life biodistribution data (see **Figure S7)**. Vehicle data is not shown for simplicity; all vehicle data are 0 copies. The same legend applies for **B-D**; and for **E-G**. LOD, limit of detection; SN, sciatic nerve.

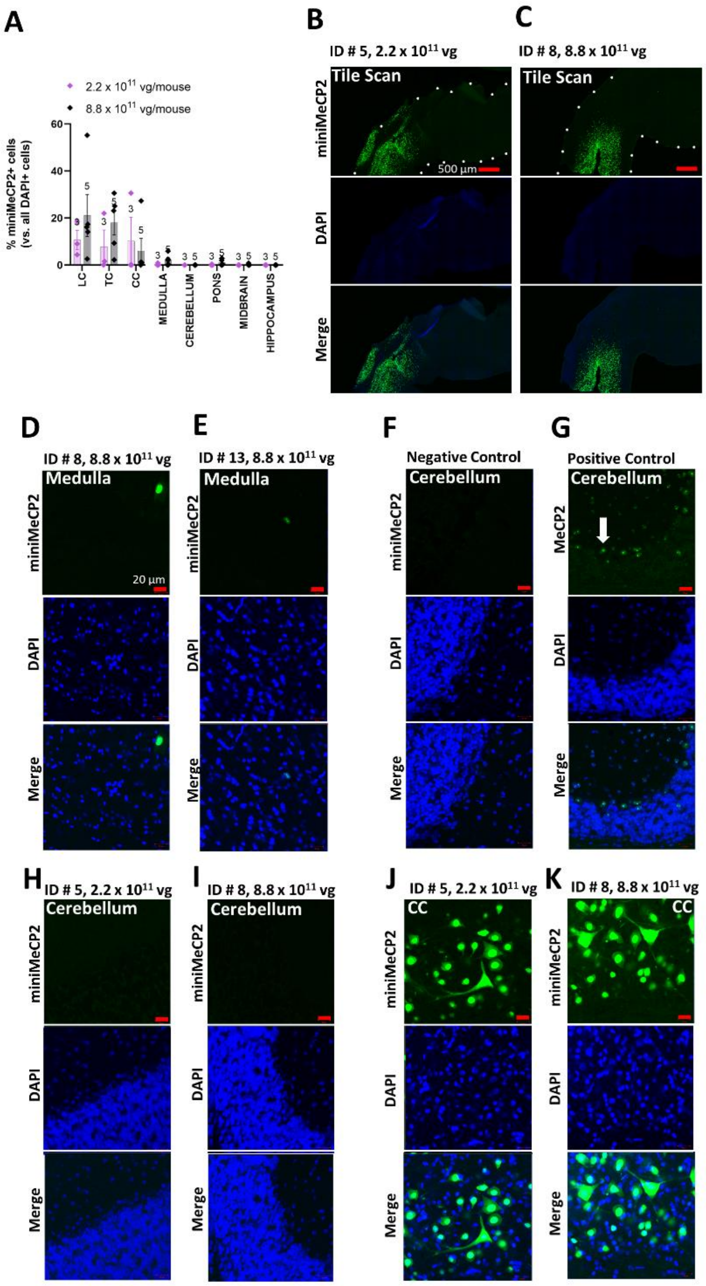

Figure S10. Expression of miniMeCP2 protein is observed primarily in the spinal cord after P38 administration in KO mice. Mice were treated at UTSWMC. (**A**) The mean percentage of miniMeCP2(+) cells versus all DAPI(+) cells for each of two KO groups enrolled. A break in the X-axis is indicated because spinal cord tissue was processed

separately from brain tissue. Data are means ± SEM. (**B-C**) Tile scans show a striking delineation in expression levels between the upper spinal cord and the medulla in TSHA-102-treated KO mice. White dots outline the tissue sections for clarity. Tile scans used consistent gain settings. (**D-E**) At higher magnification, a low percentage of miniMeCP2(+) cells can be observed in the medulla of KO mice. Mouse ID #5 had no miniMeCP2(+) cells among the 5 medullar regions sampled. (**F**) No cerebellar miniMeCP2 expression was observed in a negative control sample (KO mouse, no primary antibody). (**G**) Arrow points to a cerebellar Purkinje neuron expressing endogenous MeCP2 in the positive control. (**H-I**) No detectable miniMeCP2 protein expression in the cerebella of TSHA-102-treated KO mice. (**J-K**) Cervical spinal cord images from the same mice shown in **B-C** and **H-I**. (**B-C**) Scale bar is 500 μm. (**D-K**) Close-up images used consistent gain settings, except the gain for the blue channel (DAPI) was decreased 15% to delineate nuclei in **G**. Scale bar is 20 μm.

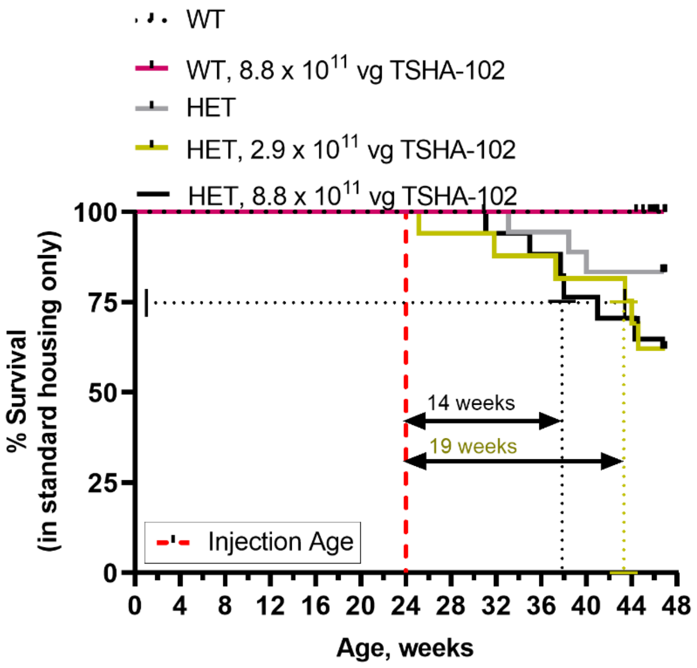

Figure S11. TSHA-102 is well-tolerated in a survival safety assessment in female WT and *Mecp2*$^{-/+}$ mice. Female RTT mice are a valuable model for assessing the safety of *MECP2* gene therapy [11]. The data above was generated at UTSWMC concurrently with efficacy studies at JAX. The same virus lot was evaluated across studies. The safety study concluded before median survivals could be identified. Depending on the dose tested, 75% of mice were still alive 14-19 weeks post-injection. No significant difference was observed between vehicle- and TSHA-102-treated *Mecp2*$^{-/+}$ mice ($p > 0.05$, n per *Mecp2*$^{-/+}$ group is 17-19 mice; 1-2 censored mice per vehicle- and TSHA-102-treated *Mecp2*$^{-/+}$ group). To avoid graphing artifacts, the end of the study is defined as 47 weeks. One high-dose *Mecp2*$^{-/+}$ mouse died at 48 weeks. The n per group is 10-23 (WT and HET groups combined). Censored mice: Each *Mecp2*$^{-/+}$ group had one veterinarian-requested euthanasia at 30-31 weeks of age. There was one veterinarian-requested euthanasia for the lower dose at nearly 45 weeks of age.

References

6. Gadalla, K.K.; Bailey, M.E.; Spike, R.C.; Ross, P.D.; Woodard, K.T.; Kalburgi, S.N.; Bachaboina, L.; Deng, J.V.; West, A.E.; Samulski, R.J.; et al. Improved survival and reduced phenotypic severity following AAV9/MECP2 gene transfer to neonatal and juvenile male Mecp2 knockout mice. *Mol Ther* **2013**, *21*, 18-30, doi:10.1038/mt.2012.200.

7. Gadalla, K.K.E.; Vudhironarit, T.; Hector, R.D.; Sinnett, S.; Bahey, N.G.; Bailey, M.E.S.; Gray, S.J.; Cobb, S.R. Development of a Novel AAV Gene Therapy Cassette with Improved Safety Features and Efficacy in a Mouse Model of Rett Syndrome. *Mol Ther Methods Clin Dev* **2017**, *5*, 180-190, doi:10.1016/j.omtm.2017.04.007.

8. Sinnett, S.E.; Boyle, E.; Lyons, C.; Gray, S.J. Engineered microRNA-based regulatory element permits safe high-dose miniMECP2 gene therapy in Rett mice. *Brain* **2021**, *144*, 3005-3019, doi:10.1093/brain/awab182.

9. Sinnett, S.E.; Hector, R.D.; Gadalla, K.K.E.; Heindel, C.; Chen, D.; Zaric, V.; Bailey, M.E.S.; Cobb, S.R.; Gray, S.J. Improved MECP2 Gene Therapy Extends the Survival of MeCP2-Null Mice without Apparent Toxicity after Intracisternal Delivery. *Mol Ther Methods Clin Dev* **2017**, *5*, 106-115, doi:10.1016/j.omtm.2017.04.006.

10. Matagne, V.; Ehinger, Y.; Saidi, L.; Borges-Correia, A.; Barkats, M.; Bartoli, M.; Villard, L.; Roux, J.C. A codon-optimized Mecp2 transgene corrects breathing deficits and improves survival in a mouse model of Rett syndrome. *Neurobiol Dis* **2017**, *99*, 1-11, doi:10.1016/j.nbd.2016.12.009.

11. Matagne, V.; Borloz, E.; Ehinger, Y.; Saidi, L.; Villard, L.; Roux, J.C. Severe offtarget effects following intravenous delivery of AAV9-MECP2 in a female mouse model of Rett syndrome. *Neurobiol Dis* **2021**, *149*, 105235, doi:10.1016/j.nbd.2020.105235.

12. Tillotson, R.; Selfridge, J.; Koerner, M.V.; Gadalla, K.K.E.; Guy, J.; De Sousa, D.; Hector, R.D.; Cobb, S.R.; Bird, A. Radically truncated MeCP2 rescues Rett syndrome-like neurological defects. *Nature* **2017**, *550*, 398-401, doi:10.1038/nature24058.

14. Garg, S.K.; Lioy, D.T.; Cheval, H.; McGann, J.C.; Bissonnette, J.M.; Murtha, M.J.; Foust, K.D.; Kaspar, B.K.; Bird, A.; Mandel, G. Systemic delivery of MeCP2 rescues behavioral and cellular deficits in female mouse models of Rett syndrome. *J Neurosci* **2013**, *33*, 13612-13620, doi:10.1523/JNEUROSCI.1854-13.2013.

15. Powers, S.; Likhite, S.; Gadalla, K.K.; Miranda, C.J.; Huffenberger, A.J.; Dennys, C.; Foust, K.D.; Morales, P.; Pierson, C.R.; Rinaldi, F.; et al. Novel MECP2 gene therapy is effective in a multicenter study using two mouse models of Rett syndrome and is safe in non-human primates. *Mol Ther* **2023**, *31*, 2767–2782, doi:10.1016/j.ymthe.2023.07.013.

16. Yang, K.; Cheng, C.; Yuan, Y.; Zhang, Y.; Shan, S.; Qiu, Z. Extension of the Lifespan of a Mouse Model of Rett Syndrome by Intracerebroventricular Delivery of MECP2. *Neurosci Bull* **2023**, *39*, 297-302, doi:10.1007/s12264-022-00974-y.

17. Sinnett, S.E.; Gray, S.J. Recent endeavors in MECP2 gene transfer for gene therapy of Rett syndrome. *Discov Med* **2017**, *24*, 153-159.
